# CD4 + T cells are found within endemic Burkitt lymphoma and modulate Burkitt lymphoma precursor cell viability and expression of pathogenically relevant Epstein–Barr virus genes

**DOI:** 10.1007/s00262-021-03057-5

**Published:** 2021-10-19

**Authors:** Semjon Sidorov, Lara Fux, Katja Steiner, Samyo Bounlom, Sabrina Traxel, Tarik Azzi, Arbeneshe Berisha, Christoph Berger, Michele Bernasconi, Felix K. Niggli, Yvonne Perner, Sugeshnee Pather, Werner Kempf, David Nadal, Simone Bürgler

**Affiliations:** 1 Experimental Infectious Diseases and Cancer Research, Children’s Research Center, University Children’s Hospital of Zurich, University of Zurich, Zurich, Switzerland; 2 Division of Anatomical Pathology, National Health Laboratory Service, Chris Hani Baragwanath Academic Hospital, School of Pathology, University of the Witwatersrand, Johannesburg, South Africa; 3 Kempf Und Pfaltz, Histological Diagnostics, Zürich, Switzerland; 4 Department of Dermatology, University Hospital Zurich, Zurich, Switzerland; 5 Department of Pediatric Hematology and Oncology, Inselspital, Bern University Hospital, University of Bern, Bern, Switzerland; 6 Department of Biomedical Research, University of Bern, Bern, Switzerland

**Keywords:** Epstein–Barr virus, T helper cells, Endemic Burkitt lymphoma, CRISPR/CAS9, Latency III to Latency I switch, *IgH/c-myc* translocation

## Abstract

Endemic Burkitt lymphoma (eBL) is an aggressive B cell cancer characterized by an *IgH/c-myc* translocation and the harboring of Epstein–Barr virus (EBV). Evidence accumulates that CD4 + T cells might contribute to eBL pathogenesis. Here, we investigate the presence of CD4 + T cells in primary eBL tissue and their potential dichotomous impact on an EBV-infected pre-eBL cell model using ex vivo material and in vitro co-cultures. In addition, we establish a novel method to study the effect of *IgH/c-myc* translocation in primary B cells by employing a CRISPR/Cas9 knock-in approach to introduce and tag de novo translocation. We unprecedently document that CD4 + T cells are present in primary eBL tumor tissue. Furthermore, we demonstrate that CD4 + T cells on the one hand suppress eBL development by killing pre-eBL cells lacking *IgH/c-myc* translocation in vitro and on the other hand indirectly promote eBL development by inducing crucial EBV Latency III to Latency I switching in pre-eBL cells. Finally, we show that while the mere presence of an *IgH/c-myc* translocation does not suffice to escape CD4 + T-cell-mediated killing in vitro, the CD4 + T-cell-mediated suppression of EBV’s Latency III program in vivo may allow cells harboring an *IgH/c-myc* translocation and additional mutations to evade immune control and proliferate by means of deregulated *c-myc* activity, resulting in neoplasia. Thus, our study highlights the dichotomous effects of CD4 + T cells and the mechanisms involved in eBL pathogenesis, suggests mechanisms of their impact on eBL progression, and provides a novel in vitro model for further investigation of *IgH/c-myc* translocation.

**Supplementary Information:**

The online version contains supplementary material available at 10.1007/s00262-021-03057-5.

## Introduction

Endemic Burkitt lymphoma (eBL), the most frequent type of Burkitt lymphoma, is widespread in sub-Saharan Africa and affects mainly immunocompetent hosts [1]. eBL is thought to originate from germinal center (GC) B cells [2] and is characterized by the presence of an *IgH/c-myc* translocation, which leads to deregulated expression of the proto-oncogene *c-myc* [3]. The *IgH/c-myc* translocation is not sufficient for eBL development, suggesting a multi-step pathogenesis [4]. Additional factors, including mutations [5] and chronic immune-stimulating co-infections such as malaria [6], are required. Importantly, 98% of eBL tumors harbor Epstein–Barr virus (EBV) [6], though its pathogenic role is incompletely elucidated.

EBV is a B-lymphotropic virus which infects more than 90% of the world’s population, usually with no ill effects [7]. Key for EBV persistence is its ability to evade the host’s immune response by entering latency in B cells. During early B cell infection, EBV is found in Latency III program. It transforms B cells into proliferating blasts and is dependent on EBV nuclear antigen 2 (EBNA2) and latent membrane protein 1 (LMP1) expression, as well as Cp promoter [8, 9]. In vitro, EBV-infected B cells remain in Latency III and proliferate almost indefinitely as lymphoblastoid cell lines (LCLs). In immunocompetent hosts, however, the immune response drives transition from Latency III to the more restricted latency programs II, I, or 0 [10], since the Latency III program is highly immunogenic [11, 12]. Thus, the vast majority of EBV-infected B cells in vivo are found in Latency I/0, during which only EBNA1, regulated by Qp promoter, is expressed [13]. EBV-infected B cells in Latency I lose proliferative capacity, but become almost undetectable by the immune system [14].

Early eBL cells are thought to derive from EBV-infected B cells that acquire *IgH/c-myc* translocation [3], probably through aberrant action of activation-induced cytidine deaminase (AID) [15], upregulated by EBV in Latency III [16]. EBV-infected eBL cells, however, harbor EBV in Latency I, with cell proliferation driven by deregulated c-myc expression due to *IgH/c-myc* translocation [3]. Therefore, Latency III to Latency I switch seems one of the key steps in eBL development, as it allows EBV-infected pre-eBL cells with an *IgH/c-myc* translocation to escape antiviral immunity. How and when this switch happens remains unclear.

The literature suggests that simultaneous expression of EBV and eBL growth programs is incompatible within the same cell [17–21]. Latency III depends on high NF-κB activity, while eBL cells usually show high c-myc activity and lack NF-κB signaling [17–21]. Furthermore, the EBV Latency III program is dominant over the *c-myc* growth program [22–25], as the presence of *IgH/c-myc* translocation alone is not enough to turn an EBV-infected B cell into an eBL cell [22, 23], and overexpression of c-myc in Latency III B cells does not induce a switch to Latency I [24, 25]. Therefore, external factors are thought to induce the Latency III-to-Latency I switch in pre-eBL cells, but their specific nature remains unknown.

There is accumulating evidence that CD4 + T cells impact on eBL pathogenesis. Several studies have suggested that CD4 + T cells act as cytotoxic killer cells against EBV-infected cells [26–29]. Other studies suggest that CD4 + T cells might support pre-eBL cells, like T follicular helper (Tfh) cell support healthy B cells, and, therefore, may inadvertently promote eBL development [30–33]. Particularly, in vitro co-culture studies have shown that at lower effector/target (E/T) ratios CD4 + T cells suppress B cell proliferation [32] by inducing Latency III to Latency I switch, characterized by loss of EBNA2 [32, 33] and LMP1 [33] expression. Lack of suitable in vitro and in vivo models, however, considerably obstacle mechanistic studies of early eBL pathogenesis and immune system contribution to eBL development.

Here, we investigated the potential pathogenic role of CD4 + T cells in eBL based on the hypothesis of a dichotomous impact of CD4 + T cells on eBL development. We hypothesize that CD4 + T cells interact with EBV-infected pre-eBL cells, similarly to Tfh cell interaction with a healthy B cell, leading to the suppression of the EBV Latency III program and reduction in EBV-infected cell outgrowth. While the switching to the BL Latency I suppresses the growth of EBV-infected B cells, it protects them from immune recognition. Moreover, we suggest that the Latency switch allows the rare *IgH/c-myc*-translocated cells to proliferate by means of deregulated *c-myc* activity, resulting in subsequent neoplasia. Thus, we aimed at engineering a novel model of pre-eBL cells harboring an *IgH/c-myc* translocation to be used in co-cultures with CD4 + T cells in vitro. Using this model, we gathered important insights into CD4 + T cells interaction with pre-eBL cells and into their potential role in early events of eBL development.

## Material and methods

All kits/reagents were used according to manufacturer’s instructions unless otherwise stated. Details on antibodies and oligos are given in Tables 1, 2 and 3.

**Table 1:** Antibody details

	Staining antibody/reagent	Supplier	Concentration (ul/100ul staining buffer)	Clone	Cat Number	Dilution	Diluted in
Flow cytometry (FC)	Human TruStain FcX	BioLegend	1.25	N/A	422,302		
	Zombie Red Viability dye	BioLegend	0.1	N/A	423,109		
	Zombie NIR Viability dye	BioLegend	0.1	N/A	423,105		
	Annexin V—APC	BioLegend	1.25	N/A	640,919		
	FxCycle Violet	Thermo Scientific	1	N/A	R37166		
	CD19—APC/Cy7	BioLegend	2.5	HIB19	302,218		
	CD19—AlexaFluor 700	BioLegend	1.25	HIB19	302,226		
	CD19—APC/Cy7	BioLegend	5	HIB19	302,218		
	CD4—BV711	Thermo Scientific	2.5	OKT4	67–0049-42		
	CD4—PerCp/Cy5.5	BioLegend	1.25	RPA-T4	300,529		
	CD40—PE/Cy7	BioLegend	2.5	5C3	334,321		
	CD40L—BV421	BioLegend	5	24–31	310,823		
	CD28—BV711	BioLegend	5	CD28.2	302,947		
	CD80—BV421	BioLegend	2.5	2D10	305,221		
	CD86—BV711	BioLegend	1.25	IT2.2	305,439		
	OX40—APC	BioLegend	5	11C3.1	326,307		
	OX40L—PE	BioLegend	2.5	ACT35	350,007		
	ICOS-L—APC	BioLegend	5	11C3.1	326,307		
	ICOS—PE/Cy7	BioLegend	5	2D3	309,407		
	FASL—PE	BioLegend	2.5	C398.4A	313,519		
	FAS—PE/Dazzle 594	BioLegend	5	NOK-1	306,406		
	HLADR—PerCp/Cy5.5	BioLegend	1.25	DX2	305,633		
	HLA A,B,C—BV510	BioLegend	5	L243	307,629		
	IFNg—APC/Cy7	BioLegend	5	W6/32	311,435		
	T-bet—BV711	BioLegend	2.5	B27	506,523		
	IL13—BV421	BioLegend	2.5	4B10	644,819		
	GATA3—Alexa 488	BioLegend	2.5	JES10-5A2	501,915		
	IL21—Alexa 647	BioLegend	2.5	16E10A23	653,807		
	bcl6—PE	BioLegend	2.5	3A3-N2	513,005		
	CD54—PerCp/eFluor 710	Thermo Scientific	5	HA58	46–0549-41		
	CD10—PE/Cy5	BioLegend	20	HI10a	555,376		
	CD23—BV421	BioLegend	5	EBVCS-5	338,521		
	CD39—BV510	BioLegend	5	A1	328,219		
FC	CD38—APC	Thermo Scientific	5	HIT2	17–0389-41		
	CD58—PE/Cy7	BioLegend	5	TS2/9	330,915		
Western Blot	Mouse anti-human EBNA2	Abcam		PE2	ab90543	1:1000	5% Milk/TBS-T
	Mouse anti-human LMP1	Abcam		CS 1–4	ab78113	1:1000	5% Milk/TBS-T
	Rabbit anti-human c-myc	Abcam		Y69	ab32072	1:2500	5% Milk/TBS-T
	Rabbit anti-human β-actin	Abcam		polyclonal	4967S	1:1000	5% Milk/TBS-T
	Rabbit anti-human β-actin	Abcam		polyclonal	4967S	1:1000	1X Blocker ™ FL Fluorescent Blocking Buffer
	Horse anti-mouse HRP (secondary)	Cell Signaling		polyclonal	7076S	1:1000	5% Milk/TBS-T
	Goat anti-rabbit HRP (secondary)	Cell Signaling		polyclonal	7074S	1:2500	5% Milk/TBS-T
	Anti-rabbit Alexa Fluor 488 (secondary)	Thermo Scientific		polyclonal	A32731	1:10,000 (Fluorescent)	1X Blocker ™ FL Fluorescent Blocking Buffer
IHC	Mouse anti-human CD3	Dako		F7.2.38		1:100	
	mouse anti-human CD8	Cell Marque		C8/144B		1:90	
	mouse anti-human CD4	Novocastra		4B12		1:80

**Table 2:** List of PCR, gDNA PCR and qRT-PCR primer/probes

	Gene/Sequence name	Direction	Sequence or assay ID	Supplier
Cloning (PCR)	c-myc PCR-1F	Forward	5'-CCGCCATCTTTAGCAACTTTC-3'	Microsynth (custom)
	c-myc PCR-1R	Reverse	5'-CACAGCAGAAGGTGATGGGTA-3'	
	IgH PCR-1F	Forward	5'-CGAGATGCCTGAACAAACCA-3'	
	IgH PCR-1R	Reverse	5'-CTTGCTTTGGCCTCAATTCC-3'	
	GFP PCR-1F	Forward	5'-CCG CGT TAC ATA ACT TAC GG-3'	
	GFP PCR-1R	Reverse	5'-AAC TTG TTT ATT GCA GCT TAT AAT GGT-3'	
gDNA PCR	gDNA nPCR-1F	Forward	5'-ATC CGC CAT CTT TAG CAA CTT T-3'	
	gDNA nPCR-1R	Reverse	5'-CTG AGC ATT GCA GGT TGG TC-3'	
	gDNA nPCR-2F	Forward	5'-AAT TCA TCT GCT TCC AGC TT-3'	
	gDNA nPCR-2R	Reverse	5'-TAT GGA GAA CCG GTA ATG GCA-3'	
	gDNA nPCR-3F	Forward	5'-TAC CCT ATG AGG TCA AGC TG-3'	
	gDNA nPCR-3R	Reverse	5'-ACA AAC CAG GGG TCT TAG TG-3'	
qRT-PCR	gDNA nPCR-4F	Forward	5'-TAT GAT GGT CAA AAC GCA GTC-3'	
	gDNA nPCR-4R	Reverse	5'-CCC CCA CGT CTT AGA AAC TC-3'	
	EBNA2	Forward	5'-GGG ATG CCT GGA CAC AAG AG-3'	
		Reverse	5'-CAT GCC CGA CGT CAT ATC CT-3'	
		Probe	FAM-5'-CATCACCTCTTGATAGGGATCCGC-3'-BHQ1	
	LMP1	Forward	5’-TGG AGC CCT TTG TAT ACT CCT-3’	
		Reverse	5’TGC CTG TCC GTG CAA ATT C-3’	
		Probe	FAM-5’-TGATCACCCTCCTGCTCATCGCTCT-3’-BHQ1	
	EBNA1 Cp	Forward	5'-TGC CTG AAC CTG TGG TTG G-3'	
		Reverse	5'-CAT GAT TCA CAC TTA AAG GAG ACG G-3'	
		Probe	FAM-5'-TCCTCTGGAGCCTGACCTGTG ATC G-3'-BHQ1	
	EBNA1 Qp	Forward	5'-GTG CGC TAC CGG ATG GC-3'	
		Reverse	5'-CAT GAT TCA CAC TTA AAG GAG ACG G-3'	
		Probe	FAM-5'-TCCTCTGGAGCCTGACCTGTGATCG-3'-BHQ1	
	bcl6		Hs.PT.56a.19673829.g (Dye: FAM)	IDT®
	c-myc		Hs.PT.58.26770695 (Dye: FAM)	IDT®
	IFNγ		Hs.PT.58.3781960 (Dye: FAM)	IDT®
	IL-13		Hs00174379_m1 (Dye: FAM)	Thermo Fisher
	IL-21		Hs.PT.58.22750196 (Dye: FAM)	IDT®
	TBX21		Hs.PT.58.3936407 (Dye: FAM)	IDT®
	GATA3		Hs.PT.58.4308511 (Dye: FAM)	IDT®
	bcl6		Hs.PT.56a.19673829.g (Dye: FAM)	IDT®
	POLR2A		Hs.PT.39a.19639531 (Dye: FAM)	IDT®
	UBC		Hs.PT.39a.22214853 (Dye: Cy5)	IDT®
	TBP		Hs.PT.39a.22214825 (Dye: FAM)	IDT®
	YWHAZ		Hs.PT.39a.22214858 (Dye: FAM)	IDT®

**Table 3:** crRNA, ssDNA, PCR primer sequences

Sequence name	Sequence (5′-3′)
crRNA IgH1	5′ AltR1-CCGUAAAAACCUACUUGACCGUUUUAGAGCUAUGCU-AltR2-3′
crRNA IgH7	5′ AltR1-CCGAAGGAUCGCUUUAACCGGUUUUAGAGCUAUGCU-AltR2-3′
crRNA IgH10	5′ AltR1-CCUCGGUUAAAGCGAUCCUUGUUUUAGAGCUAUGC-AltR2-3′
crRNA c-myc4	5′ AltR1-GGACGGCAGCCACCGUUUCUGUUUUAGAGCUAUGCU-AltR2-3′
crRNA c-myc5	5′ AltR1-GCCGUCCUGACAGGGGCUUAGUUUUAGAGCUAUGCU-AltR2-3′
crRNA c-myc6	5′ AltR1-GCCCCGGUCUUGAUGAGAGCGUUUUAGAGCUAUGCU-AltR2-3′
GFP PCR-1F	5′-CCG CGT TAC ATA ACT TAC GG-3′
GFP PCR-1R	5′-AAC TTG TTT ATT GCA GCT TAT AAT GGT-3′
STR1-F	5′ GGC TTC TGA GGC GGA AAG′
STR1-R	5′ CTT TTT ACG GTT CCT GGC CTT′
IgH gene block	5′cggaaagaaccagcGGCGCGAGTGCCAGATTCCTGGGAAATCAGCCTACAAGGCTCCTGCGGGAAGGAACCTCCACTGCCAGAAGTCCTTAGGGCATCTAAGTGATCAGACACCGTCAGGGATTCTTTGCCCCGTAAAAACCTACTTGACCAGGGACACGTGCCAGGTAAATTTCCTTCACATTTACTTCAACCTTATTGCATACTCATTTTAGTATTAAAACCTTTAATAAAATGCTCCTATTCCTTCACACTTTTTTTCTATGAGATCTCAAATACCCCTTCTTGCTATTAAAAAAAATCACTTATTATTCACCAGCCCAATATTTTAAAAGTAAAAATAATAAGCCAAGGCCAGGAGCGATGACTCGCACTTGTATTCCCAGCAGTTTCAGAGGCAAAGGCCGAAGGATCGCTTTAACCGCGTTACATAACTTACGG-3′
c-myc gene block	5′TAAGCTGCAATAAACAAGTTCTCATCAAGACCGGGGCTACGCGTCCCTCCTGGCTGGATTCACCCACTCCGACAGTTCTCTTTCCAGCCAATAAAGAATTTAAGATGCAGGTTGACACACAGCGCACCTCATAATTCTAAAGAAAATATTTCACGATTCGCTGCTGTGCAGCGATCTTGCAGTCCTACAGACACCGCTCCTGAGACACATTCCTCAGCCATCACTAAGACCCCTGGTTTGTTCAGGCATCTCGTCCAAATGTGGCTCCCCAAGCCCCCAGGCTCAGTTACTCCATCAGACGCACCCAACCTGAGTCCCATTTTCCAAAGGCATCGGAAAATCCACAGAGGCTCCCAGATCCTCAAGGCACCCCAGTGCCCATCCCCTCCTGGCCAGTCCGCCCAGGTCCCCTCGGAACATCTAGCcaaaaggccaggaac-3′
GFP + CMV ssDNA	5′GGCTTCtgaggcggaaagaaccagcGGCGCGAGTGCCAGATTCCTGGGAAATCAGCCTACAAGGCTCCTGCGGGAAGGAACCTCCACTGCCAGAAGTCCTTAGGGCATCTAAGTGATCAGACACCGTCAGGGATTCTTTGCCCCGTAAAAACCTACTTGACCAGGGACACGTGCCAGGTAAATTTCCTTCACATTTACTTCAACCTTATTGCATACTCATTTTAGTATTAAAACCTTTAATAAAATGCTCCTATTCCTTCACACTTTTTTTCTATGAGATCTCAAATACCCCTTCTTGCTATTAAAAAAAATCACTTATTATTCACCAGCCCAATATTTTAAAAGTAAAAATAATAAGCCAAGGCCAGGAGCGATGACTCGCACTTGTATTCCCAGCAGTTTCAGAGGCAAAGGCCGAAGGATCGCTTTAACCGCGTTACATAACTTACGGTAAATGGCCCGCCTGGCTGACCGCCCAACGACCCCCGCCCATTGACGTCAATAATGACGTATGTTCCCATAGTAACGCCAATAGGGACTTTCCATTGACGTCAATGGGTGGAGTATTTACGGTAAACTGCCCACTGGCAGTACATCAAGTGTATCATATGCCAAGTCCGCCCCCTATTGACGTCAATGACGGTAAATGGCCCGCCTGGCATTATGCCCAGTACATGACCTTACGGGACTTTCCTACTTGGCAGTACATCTACGTATTAGTCATCGCTATTACCATGGTGATGCGGTTTTGGCAGTACACCAATGGGCGTGGATAGCGGTTTGACTCACGGGGATTTCCAAGTCTCCACCCCATTGACGTCAATGGGAGTTTGTTTTGGCACCAAAATCAACGGGACTTTCCAAAATGTCGTAATAACCCCGCCCCGTTGACGCAAATGGGCGGTAGGCGTGTACGGTGGGAGGTCTATATAAGCAGAGGTCGTTTAGTGAACCGTCAGATCACTAGTAGCTTTATTGCGGTAGTTTATCACAGTTAAATTGCTAACGCAGTCAGTGCTCGACTGATCACAGGTAAGTATCAAGGTTACAAGACAGGTTTAAGGAGGCCAATAGAAACTGGGCTTGTCGAGACAGAGAAGATTCTTGCGTTTCTGATAGGCACCTATTGGTCTTACTGACATCCACTTTGCCTTTCTCTCCACAGGGGTACCGAAGCCGCTAGCGCTACCGGTCGCCACCATGCCCGCCATGAAGATCGAGTGCCGCATCACCGGCACCCTGAACGGCGTGGAGTTCGAGCTGGTGGGCGGCGGAGAGGGCACCCCCGAGCAGGGCCGCATGACCAACAAGATGAAGAGCACCAAAGGCGCCCTGACCTTCAGCCCCTACCTGCTGAGCCACGTGATGGGCTACGGCTTCTACCACTTCGGCACCTACCCCAGCGGCTACGAGAACCCCTTCCTGCACGCCATCAACAACGGCGGCTACACCAACACCCGCATCGAGAAGTACGAGGACGGCGGCGTGCTGCACGTGAGCTTCAGCTACCGCTACGAGGCCGGCCGCGTGATCGGCGACTTCAAGGTGGTGGGCACCGGCTTCCCCGAGGACAGCGTGATCTTCACCGACAAGATCATCCGCAGCAACGCCACCGTGGAGCACCTGCACCCCATGGGCGATAACGTGCTGGTGGGCAGCTTCGCCCGCACCTTCAGCCTGCGCGACGGCGGCTACTACAGCTTCGTGGTGGACAGCCACATGCACTTCAAGAGCGCCATCCACCCCAGCATCCTGCAGAACGGGGGCCCCATGTTCGCCTTCCGCCGCGTGGAGGAGCTGCACAGCAACACCGAGCTGGGCATCGTGGAGTACCAGCACGCCTTCAAGACCCCCATCGCCTTCGCCAGATCTCGAGCTCGATGAGTTTGGACAAACCACAACTAGAATGCAGTGAAAAAAATGCTTTATTTGTGAAATTTGTGATGCTATTGCTTTATTTGTAACCATTATAAGCTGCAATAAACAAGTTCTCATCAAGACCGGGGCTACGCGTCCCTCCTGGCTGGATTCACCCACTCCGACAGTTCTCTTTCCAGCCAATAAAGAATTTAAGATGCAGGTTGACACACAGCGCACCTCATAATTCTAAAGAAAATATTTCACGATTCGCTGCTGTGCAGCGATCTTGCAGTCCTACAGACACCGCTCCTGAGACACATTCCTCAGCCATCACTAAGACCCCTGGTTTGTTCAGGCATCTCGTCCAAATGTGGCTCCCCAAGCCCCCAGGCTCAGTTACTCCATCAGACGCACCCAACCTGAGTCCCATTTTCCAAAGGCATCGGAAAATCCACAGAGGCTCCCAGATCCTCAAGGCACCCCAGTGCCCATCCCCTCCTGGCCAGTCCGCCCAGGTCCCCTCGGAACATCTAGCcaaaaggccaggaaccgtaaaAAG-3′
pmax-GFP plasmid	http://www.addgene.org/vector-database/3525/
pcDNA3.1	https://www.addgene.org/vector-database/2093/

### Patient material

The study was conducted according to the Helsinki Declaration 1964 and approved by the regional ethics committees (Kantonale Ethikkommission Zürich, KEK-ZH Nr. StV 40/05, and University of the Witwatersrand, Johannesburg, South Africa, Nr. M180294). Human palatine tonsils were obtained from children undergoing routine tonsillectomy at the University Children’s Hospital Zurich after informed parental consent. Thirteen archived cases of BL were retrieved from the Division of Anatomical Pathology, National Health Laboratory Service, Chris Hani Baragwanath Academic Hospital, School of Pathology, University of the Witwatersrand, Johannesburg, South Africa. Tissue samples were fixed in 10% neutral buffered formalin. Ten recuts per patient were performed from paraffin tissue blocks and shipped to Zurich for immunohistochemistry (IHC). For additional patient information, see Supplementary Table 1.

### Cell culture

Cells were cultured in cRPMI medium, prepared as described in [40]. For co-culture experiments, cRPMI media was supplemented with 20U/ml of human recombinant IL-2 (Roche), referred as co-culture media.

### Isolation of tonsillar mononuclear cells from tonsils

Tonsillar mononuclear cells (TMCs) were isolated from fresh tonsils as described [34].

### Preparation of EBV virus stock and generation of lymphoblastoid cell lines

Concentrated EBV virus stock was prepared using B95-8/ZHT cell line (kind gift from Micah Luftig) as described [35] and used for generation of lymphoblastoid cell lines (LCLs) from TMCs [34].

### Isolation and expansion of CD4 + T cells

CD4 + T cells were isolated from TMCs using the CD4 MicroBeads and the autoMACS Pro Separator (Miltenyi Biotec). CD4 + T cells were polyclonally expanded for 7–10d using CD3/CD28 beads (Dynabeads® Human T-Activator CD3/CD28, Gibco by Thermo Fisher Scientific). Prior to co-culture, CD4 + T cells were rested by withdrawal of IL-2 and CD3/CD28 beads for 24–48 h.

### Co-culture setup

Co-cultures between autologous LCLs and CD4 + T cells were set up in 6-well flat-bottom plates (Sarstedt) in 3 ml of co-culture media/well at 1.5mio/well total cell density. Anti-CD3/CD28 beads were used to stimulate T cells in co-culture, unless stated “rested.” For 4:1 T cell/LCL cell ratio, 1.2mio/well CD4 + T cells and 0.3mio/well LCL were initially plated. For 1:1 T cell/LCL ratio, 0.75mio/well of both CD4 + T cells and LCL were plated. For control conditions, CD4 + T cells and LCL were cultured alone at corresponding cell densities. Cells were co-cultured for up to 9d, split every second day.

### Post-co-culture separation of LCLs and CD4 + T cells

LCLs and CD4 + T cells from co-culture were separated using CD19 MicroBeads and autoMACS Pro Separator (both Miltenyi Biotec). Purity post-separation was determined using flow cytometry. Cells were then lysed for protein and RNA isolation.

### Flow cytometry

For all stainings, cells were treated with Zombie viability dye and Fc receptor blocker and stained with anti-CD19 and anti-CD4 gating antibodies (Table 1). For surface marker stainings, cells were then stained with a corresponding panel of target-specific or isotype antibodies, washed and immediately acquired by flow cytometer. For apoptosis assay, after the surface staining cells were washed twice in Annexin V binding buffer (10 mM HEPES, 140 mM NaCl, 5 nM CaCl_2_), they were stained with anti-human Annexin V antibody for 15 min on ice and then immediately acquired by flow cytometer. EdU incorporation assay was performed using Click-iT™ Plus EdU Alexa Fluor 647 Flow Cytometry Assay Kit (Invitrogen). For cell cycle analysis, FxCycle™ dye (Thermo Scientific) was added 30 min prior to acquisition by flow cytometer. Cytokine/transcription factor staining was performed using Foxp3/Transcription Factor Staining Buffer Kit (eBioscience by Thermo Scientific). Briefly, cytokine production in co-culture was induced by adding 1 μg/ml PMA (Abcam) and 1 μg/ml Ionomycin (Abcam) for 4 h before harvesting. Brefeldin A at 5 μg/ml (Abcam) was added for 3 h prior to harvesting to induce cytokine accumulation in the cell. After the surface and viability stainings, cells were fixed, permeabilized and then stained with a corresponding panel of target-specific or isotype intracellular antibodies (see Table 1). BD LSRFortessa™ (BD Biosciences) with FACSDiva™ software was used for data acquisition; FlowJo 10.6 software was used for post-acquisition analysis.

### Western blotting

For total protein extraction, cells were lysed in RIPA buffer (Sigma-Aldrich) with protease inhibitor cocktail (cOmplete Tablet mini, Roche Diagnostics) and sonicated using an EpiShear™ Probe Sonicator (Active Motif) at 40% amplitude for 2 s. Protein concentration was measured using Pierce™ 660 nm Protein Assay Kit (Pierce by Thermo Scientific). Proteins were separated on 4–12% NuPage Gels (Invitrogen by Thermo Scientific) and transferred to either nitrocellulose membranes (GE Healthcare Life Science) or PVDF membranes (Thermo Scientific). Nitrocellulose membranes were blocked with 5% nonfat dry milk and used for detection with HRP secondary antibodies. PVDF membranes, blocked with 1X Blocker ™ FL Fluorescent Blocking Buffer (Thermo Scientific) and fluorescent secondary antibodies for β-actin were used. HRP signal was detected using Amersham ECL™ Western Blotting Reagent (GE Healthcare Life Science). ChemiDoc Imaging System (Bio-Rad Laboratories) was used to acquire both HRP and fluorescent signal. Band intensity (volume) for proteins of interest was quantified using ImageLab 6 (Bio-Rad Laboratories) software and normalized to loading control (β-actin).

### qRT-PCR

RNA was isolated using either Quick-RNA MiniPrep Kit (Zymo Research) with additional DNase treatment (ezDNase™, Thermo Scientific). RNA concentration was measured using NanoDrop (Thermo Scientific). cDNA was generated using High-Capacity cDNA Reverse Transcription Kit (Thermo Scientific). Gene expression was measured using either pre-designed TaqMan assays (IDT) or in-house designed primer–probe mixes (synthesized by Microsynth). Primer/probe sequences for Cp and Qp promoter usage assay were obtained from [36]. qRT-PCR was performed on a CFX384 Touch Real-Time PCR Detection System (Bio-Rad Laboratories AG) or 7900HT Fast Real-Time PCR System (Thermo Fisher Scientific). LCL gene expression was normalized to the geometric mean of 2 endogenous controls (TBP and YWHAZ) using dCt method. Endogenous controls were selected based on stability pre-validation using qbase 3.2 software.

### Fluorescent in situ hybridization (FISH)

In total, 100 000 LCLs were attached to microscopy slides using Cytospin funnels (500 rpm for 5 min at RT), fixed with 70% ethanol (15 min at 4C) and stored at -20C until the day of the analysis.

2.5 μl of *IgH/c-myc* translocation dual fusion probe (LPH-041, Cytocell) was added per slide, and slides were incubated in thermal cycler (2 min at 75C then at 37C overnight). Next day, slides were washed with 0.4xSSC (2 min at 72C) and 2xSSC + 0.05% Tween-20 (1 min at RT). Samples were then counterstained with DAPI, and images were acquired using Metafer Slide Scanning Platform (MetaSystems).

### Immunohistochemistry (IHC)

Biopsy specimens were fixed in 10% buffered formalin, embedded in paraffin and stained with hematoxylin and eosin (H&E). The immunohistochemical stains were conducted using a Leica BOND-MAX stainer (Leica Microsystems). Antigen retrieval included the application of ER2 solution (Leica Microsystems). Sections of human tonsils were used as positive controls.

### Evaluation of the IHC slides and data analysis

All slides were evaluated by one of the coauthors (W.K.) blinded to clinical data. Total cell number of the biopsy was estimated by counting cells based on hematoxylin and eosin staining, and the number of marker-positive cells was estimated by counting marker-positive cells. The results were given in percentage of the whole number of cells compared to the entire infiltrate and scored as (0) negative, (1) < 1%, (2) 1–5%, (3) 6–10%, (4) 11–20%, (5) 21–30%. Individual scores can be found in Supplementary Fig. 2. Representative images were taken with Axio Observer Z1 (Zeiss) and Digital Camera ORCA Flash 4.0 (Hamamatsu) using TL Brightfield channel. Images were not edited except of adjustment of Black/White intensity.

### Generation of *IgH/c-myc +* LCLs using CRISPR/CAS9 approach

crRNAs for *IgH* and *c-myc* regions were designed using the online tool (www.crispr.mit.edu). CAS9 ribonuclear protein complexes (RNPs) were formed in vitro from specific crRNAs (synthesized by IDT), standardized tracrRNA (Alt-R CRISPR-CAS9 tracrRNA, IDT) and CAS9 protein (Alt-R S.p. CAS9 Nuclease V2, IDT). Then, 200 000 LCLs were electroporated with CAS9 RNPs (1.5 μM each), ssDNA template (1 μg/transfection) and 1.8 μM of transfection enhancer (IDT) in 10 μl tips using Neon Transfection System (Thermo Scientific) at 1400 V, 10 ms, 3 pulses. Electroporated cells were outgrown for 2 weeks, and GFP + cells were flow-sorted using FACSAria Fusion cell sorter (BD Biosciences). Cells were grown for 2 additional weeks, and purity was regularly checked by monitoring percentage of GFP + cells by flow cytometer. Additional sorts were performed if percentage of GFP + cells dropped significantly.

### Generation of ssDNA for GFP knock-in

ssDNA insert was designed to consist of GFP driven by CMV promoter, flanked by two 400 bp regions homologous to *IgH* and *c-myc* regions near to breakpoint. *IgH* and *c-myc* homology regions were added in order to facilitate specific integration of GFP + CMV insert into reciprocal translocation breakpoint. ssDNA insert was generated using Guide-it Long ssDNA production system (Takara Bio). Briefly, we assembled 400 bp homology regions (ordered as GeneBlocks from IDT), GFP + CMV insert (amplified by PCR from pmax-GFP plasmid (Amaxa, Lonza) using GFP-PCR1 primers) and backbone from pcDNA3.1 plasmid (Invitrogen, Thermo Scientific) using In-Fusion HD Cloning Plus kit (Takara Bio). Then, we generated PCR product using Phusion High-Fidelity DNA polymerase (Thermo Scientific) and STR1 primers and digested sense strand of PCR using Strandase enzyme (from ssDNA production system). ssDNA was then isolated using NucleoSpin Gel and PCR Clean-Up Kit (Takara Bio).

### gDNA PCR

Genomic DNA (gDNA) was isolated using DNAeasy Blood and Tissue Kit (Qiagen). DNA concentration was determined by NanoDrop (Thermo Scientific). PCR was performed using Phusion High-Fidelity DNA polymerase (Thermo Scientific). The second round of nested PCR was performed by diluting product of first-round PCR 1:100 in ddH_2_0.

### Statistical analysis and graph design

Statistical analysis was performed using GraphPad Prism 8.4.3 (GraphPad Software). Graphs and drawings were created using GraphPad Prism 8.4.3 (GraphPad Software) and PowerPoint 2019 (Microsoft Corporation).

## Results

### CD4 + T cells are present in primary eBL tissue

**Fig. 1:**
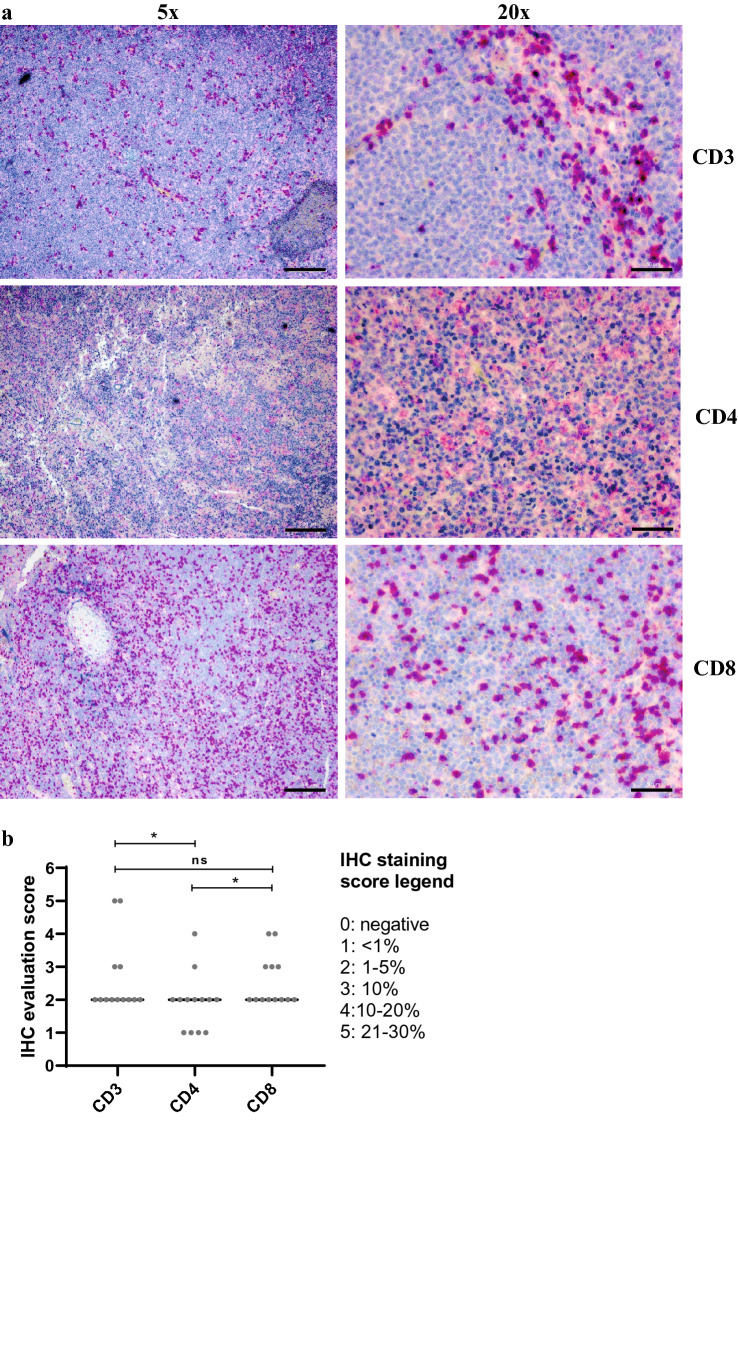
CD4 + T cells are present in primary eBL tissue. Recuts from 13 eBL patient biopsy samples were stained for CD3, CD4 and CD8 markers. **a** Images of CD3, CD4 and CD8 stainings from one representative eBL patient (BL13) at 5 × or 20 × resolution. At 5 × resolution measure bar is 200 μm, at 20 × resolution measure bar is 50 μm for all images. **b** Mean IHC evaluation score for 13 patients used for the analysis. *p* values were calculated using paired one-way ANOVA with Sidak’s test for multiple comparisons. *p* > 0.05 not significant (n.s.), *p* < 0.1*

Given that the presence of CD4 + T cells in primary eBL tumors has not been reported before, we assessed the presence and frequency of T cells in eBL tissue from 13 patients using IHC. CD3 + , CD4 + and CD8 + cells were present in all eBL samples, mostly at similar frequencies (1–5% of total cells), independent of tumors’ anatomic origin (Fig. 1b). However, CD3 + and CD8 + cells appear to be more numerous as compared to CD4 + cells (Fig. 1a and b).

The above results unprecedentedly show that CD4 + T cells are present in primary eBL tumor tissue and thus likely are present at the site of eBL development, suggesting that they may contribute to eBL pathogenesis.

### CD4 + T cells suppress pre-eBL cell outgrowth in vitro by reducing their viability

Having demonstrated the presence of CD4 + T cells in primary eBL tissue, we addressed their possible impact on pre-eBL cells. To this end, we set up co-cultures of polyclonal aCD3/CD28 beads-activated CD4 + T cells with autologous LCLs and assessed LCL proliferation, viability and cell cycle phases using flow cytometry. We chose LCLs to model pre-eBL EBV-infected B cell for two reasons: First, EBV in LCLs is in Latency III, as it is in pre-eBL cells prior to acquiring the *IgH/c-myc* translocation; and second, LCLs derived from tonsils allow setting up co-cultures with autologous CD4 + T cells. We used polyclonally activated CD4 + T cells rather than EBV-specific CD4 + T cell lines, since this allows working with a heterogeneous pool of CD4 + T cells, as expected to be found in lymph nodes/tonsils during eBL initiation. Moreover, polyclonal stimulation may mimic chronic stimulation of CD4 + T cells as in chronic malaria with which eBL is tightly associated [37].

**Fig. 2:**
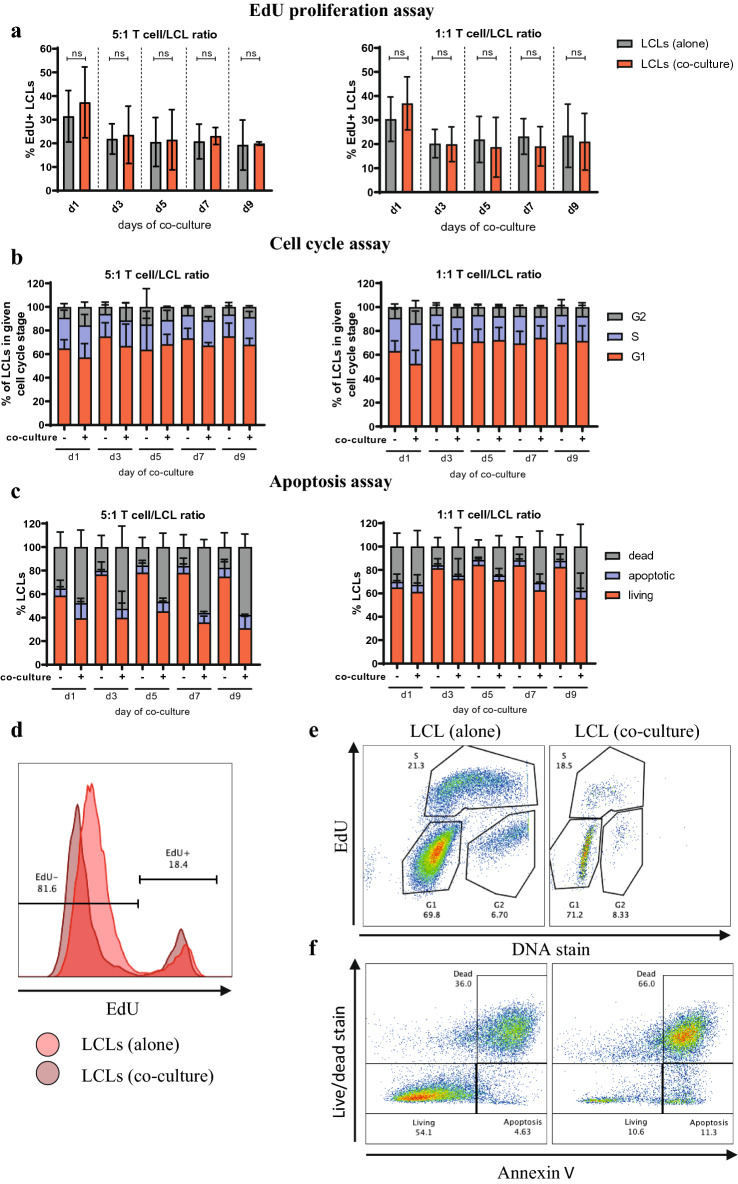
Effect of CD4 + T cells on LCL proliferation, cell cycle and viability. LCLs were cultured for 9 days either alone or in co-culture at various ratios with expanded autologous CD4 + T cells activated using anti-CD3/CD28 beads. At given times cells were harvested, stained and analyzed using flow cytometry. LCLs were pre-gated based on CD19 expression. Shown are mean ± SD of mean percentage of positive cells from 3 TMC donors **a** Mean percentage of EdU + LCLs was measured using Click-iT Flow Cytometry kit. *p* values were calculated using two-way ANOVA with Sidak’s test for multiple comparisons. *p* > 0.05 not significant (n.s.) **b** Mean percentage of LCLs in different cell cycle stages was measured using EdU Click-iT Flow cytometry kit and FxCycle dye. **c** Mean percentage of dead, apoptotic and living LCLs was measured using Annexin V stain and Zombie Viability stain. **d** Histogram overlay used for gating on EdU + and EdU- cells from one representative donor. **e** Pseudocolor plot used for gating on cells in G1, G2 and S phases of cell cycle for cell cycle assay from one representative donor. **f** Pseudocolor plot used for gating on living, apoptotic and dead cells for apoptosis assay from one representative donor

In contrast to previous reports [32], we could not detect any effect of CD4 + T cells on LCL proliferation (Fig. 2a, d), nor on the relative percentage of LCLs in different T cell cycle phases (Fig. 2b, e) at any of the time points assessed, strongly suggesting that CD4 + T cells have no effect on LCL proliferation in our co-culture model. However, we observed a markedly reduced viability of LCLs proportionally to the number of CD4 + T cells used, while the proportion of apoptotic cells was longitudinally not affected (Fig. 2c, f).

These results suggest that CD4 + T cells suppress pre-eBL cell outgrowth by reducing their viability, rather than by affecting their proliferation.

### CD4 + T cells induce switch toward eBL phenotype in pre-eBL cells

**Fig. 3:**
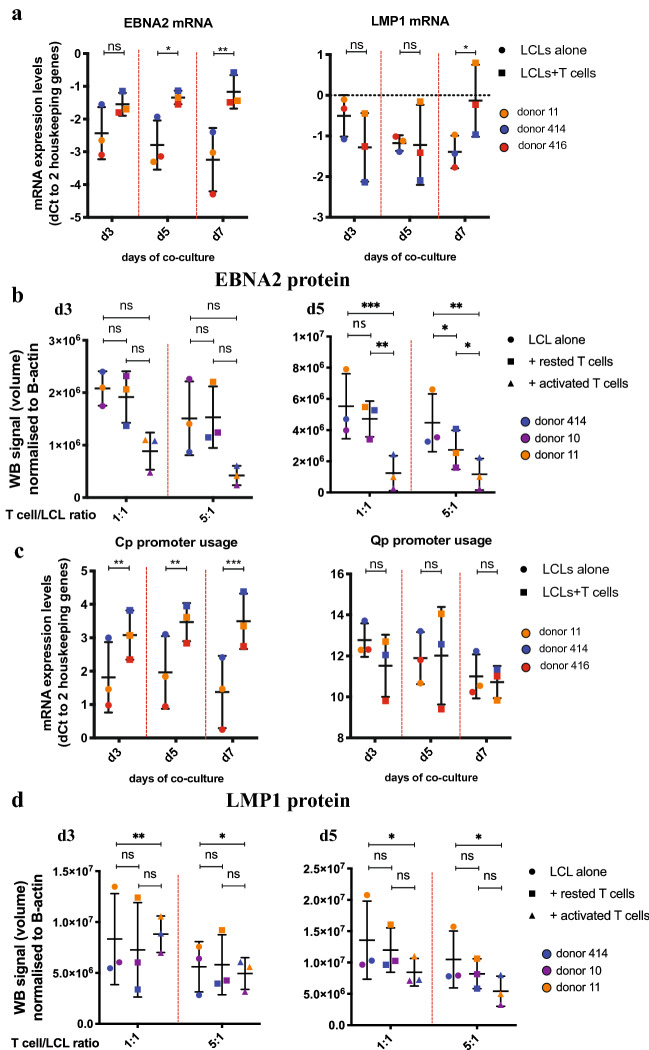
Effect of CD4 + T cells on LCL EBV Latency stage and EBV gene expression. LCLs were cultured for 7 days either alone or in co-culture at various ratios with expanded autologous CD4 + T cells activated using anti-CD3/CD28 beads. At given time points, cells were harvested and LCLs were isolated using CD19 + beads and autoMACS. Different symbols represent different conditions, while different colors represent different TMC donors. *p* values were calculated using two-way ANOVA with Sidak’s test for multiple comparisons. *p* > 0.05 not significant (n.s.), *p* < 0.1*, *p* < 0.01**, *p* < 0.001***. WB images used for quantification can be found in Supplementary Fig. 1. **a** *EBNA2* and *LMP1* expression at 5:1 T cell/LCL ratio was determined using qRT-PCR. Shown are mean ± SD of dCt values normalized to geometric means of *TBP* and *YWHAZ*. **b** Total *EBNA2* protein expression was assessed using Western blotting. Western blot image was quantified and normalized to *β-actin* as loading control. Shown are mean ± SD of normalized volume of *EBNA2* band. **c** Cp and Qp promoter usage at 5:1 T cell/LCL ratio was determined using qRT-PCR. Shown are mean ± SD of dCt values normalized to geometric means of *TBP* and *YWHAZ.* **d** Total *LMP1* protein expression was assessed using Western blotting. Western blot image was quantified and normalized to β-actin as loading control. Shown are mean ± SD of normalized volume of *LMP1* band

As CD4 + T cells reduced the viability of pre-eBL cells in our in vitro system, we hypothesized that CD4 + T cells may promote eBL development indirectly by suppressing the immunogenic EBV Latency III program, allowing pre-eBL cells to escape immune control. To test this, we analyzed changes in expression of several crucial EBV and BL markers in LCLs after co-culture with autologous CD4 + T cells. CD4 + T cells suppressed EBNA2 expression on both mRNA (Fig. 3a) and protein (Fig. 3b, Supplementary Fig. 1) levels and reduced Cp promoter usage in LCLs (Fig. 3c). Furthermore, activated CD4 + T cells induced stronger suppression of EBNA2 protein than non-activated T cells (Fig. 3b). Notably, there was no effect of CD4 + T cells on LMP1 mRNA (Fig. 3a) or protein (Fig. 3d, Supplementary Fig. 1) levels nor on Qp promoter usage (Fig. 3c). This suggests that while CD4 + T cells initiate switch from the dominant Latency III toward Latency I by decreasing EBNA2 expression, the switch is not complete as in eBL, where LMP1 expression is absent. Furthermore, while we did not observe any effect of CD4 + T cells on *c-myc* expression levels (Supplementary Figs. 1, 10), CD4 + T cells upregulated *bcl6* mRNA expression in LCLs (Supplementary Fig. 10). As *bcl6* is one of the key markers of eBL, this further supports the notion that CD4 + T cells initiate phenotypic changes in LCLs leading to eBL.

Overall, these data suggest that while CD4 + T cells do not directly promote pre-eBL cell outgrowth, by reducing EBNA2 expression they initiate a switch of EBV Latency toward an eBL-like phenotype. This, in turn, may promote the switch of cells with eBL-associated mutations and translocations to the EBV Latency I program through suppression of dominant EBV phenotype Latency III.

### CD4 + T cells can interact with pre-BL cells via both contact-dependent and soluble mediators

**Fig. 4:**
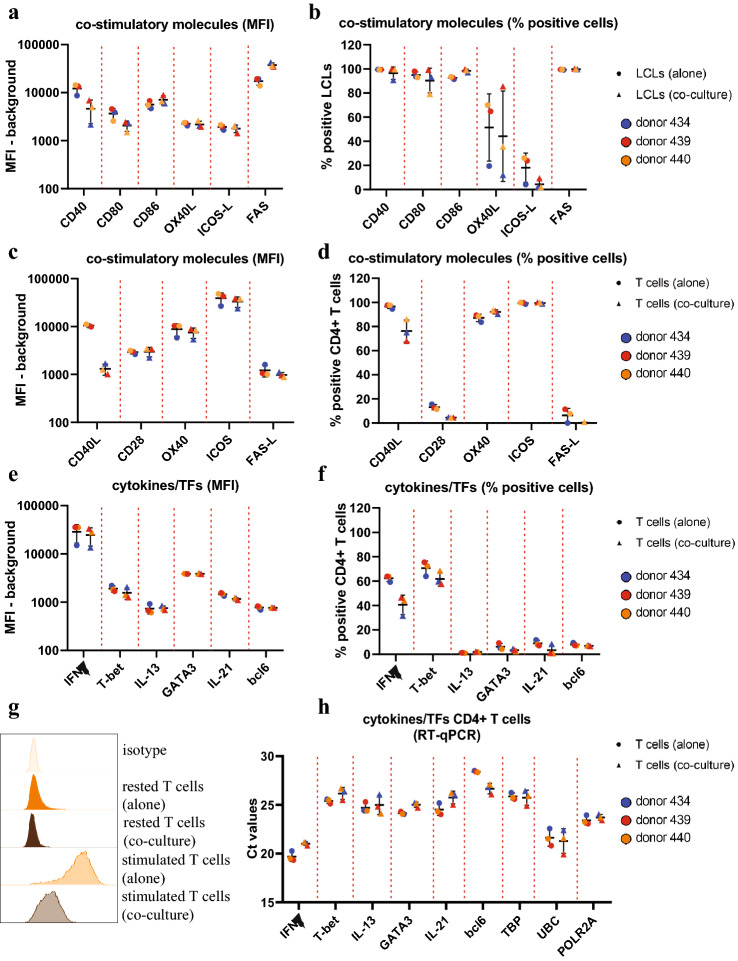
Expression of co-stimulatory molecules and T helper subset-associated markers in in vitro T cell–LCL co-culture system. LCLs were cultured either alone or in co-culture with 1:3 LCL/T cell ratio of expanded autologous CD4 + T cells activated using anti-CD3/CD28 beads. CD4 + T cells were cultured alone either activated with beads or rested as a control. After 5 days of co-culture, cells were harvested, stained and analyzed using flow cytometer. LCLs were pre-gated based on CD19 expression. T cells were pre-gated based on CD4 expression. Isotype staining was used to determine positive cells. Shown are mean ± SD of either mean fluorescent intensity (MFI) or percentage of positive cells. Different symbols represent different conditions, while different colors represent different TMC donors. Histogram overlays for one representative donor can be found in Supplementary Fig. 2. **a**, **b** Mean fluorescent intensity (MFI) (**a**) and percentage of positive cells (**b**) for expression of surface co-stimulatory molecules on LCLs. **c**, **d** Mean fluorescent intensity (MFI) **(c)** and percentage of positive cells (**d**) for expression of surface co-stimulatory molecules on CD4 + T cells. **e**, **f** Mean fluorescent intensity (MFI) (**e**) and percentage of positive cells (**f**) for cytokine production and transcription factor expression in CD4 + T cells. **g**. Histogram overlay for CD40L expression for one representative donor. **h** After 5 days of co-culture, CD4 + T cells were isolated by negative selection using CD19 + beads and autoMACS. Gene expression was determined using qRT-PCR. Shown are mean ± SD of dCT values normalized to geometric means of *POL2A*, *UBC* and *TBP*

Next, we wanted to gain information about the possible mechanisms underlying the changes in viability and gene expression observed in LCLs upon co-incubation with autologous CD4 + T cells. To this end, we first assessed the expression of several co-stimulatory molecules pairs, known to be involved in antigen-presenting cell (APC)–T cell interaction [38], using flow cytometry. LCLs in co-culture with CD4 + T cells expressed high levels of CD80/86 and CD40 as well as lower but detectable levels of ICOS-L and OX40L (Fig. 4a, 4b, Supplementary Figure S2a), while CD4 + T cells expressed OX40, CD40L and ICOS as well as low levels of CD28 (Fig. 4c, 4d, Supplementary Figure S2b). Thus, both the CD4 + T cells and the LCLs expressed their part of the co-stimulatory pairs of the four pathways we investigated, supporting the notion that CD4 + T cells can affect the phenotype of LCL through conventional T–B cell interaction pathways. Furthermore, we could observe a downregulation of CD40L on CD4 + T cells upon co-culture with LCLs (Fig. 4g), indicating an active CD40L–CD40 interaction.

Secondly, we assessed the CD4 + T helper subset composition and CD4 + T cell-derived cytokines in co-culture using both flow cytometry and qRT-PCR. We observed a high proportion of Th1 cells (T-bet + , IFNγ +), but no Th2 cells (GATA3 + , IL-13 +) or Tfh cells (bcl6 + , IL-21 +) by flow cytometry (Fig. 4e, 4f, Supplementary Figure S2c). Furthermore, we detected IL-21 and IL-13 on mRNA level (Fig. 4h), but no intracellular protein by flow cytometry (Fig. 4e, 4f). IFNγ, in contrast, was highly expressed at both mRNA and protein level.

Collectively, these data suggest that CD4 + T cells in co-culture are predominantly of Th1 phenotype and that IFNγ and CD40L are likely to be involved in CD4 + T-cell-mediated changes in LCL phenotype.

### Establishment and validation of a novel model of *IgH/c-myc* translocation and knock-in of a GFP tag in pre-BL cells using CRISPR/CAS9

**Fig. 5:**
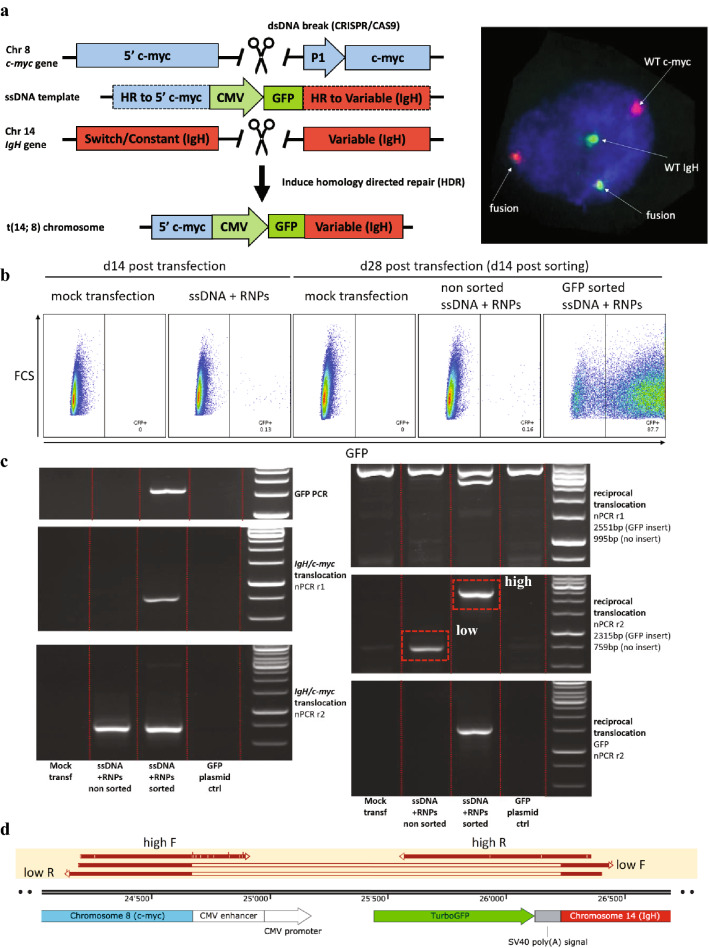
Generation and validation of *IgH/c-myc* + LCLs. **a** Overall experimental setup for generation of *IgH/c-myc* + LCLs. **b-e** LCL line was electroporated with *IgH/c-myc* targeting CAS9 RNPs and with ssDNA template containing the CMV-GFP insert. Cells were outgrown for 14 days, and then GFP + cells were sorted using FACS. GFP signal and presence of the translocations of interest were assessed using flow cytometry (**b**) and nested PCR (**c**). Highlighted PCR bands from nPCR assay were sequenced to confirm identity of the bands (**d**). Presence of *IgH/c-myc* translocation was confirmed by dual-probe FISH (**e**). Repeats performed on additional donors can be found in Supplementary Fig. 5

In order to mechanistically test whether suppression of the EBV Latency III by CD4 + T cells allows pre-eBL cells with eBL-associated genetic abnormalities to switch to the Latency I program, we modeled the main eBL-associated genetic abnormality, i.e., the *IgH/c-myc* translocation. For this, we generated LCLs from four healthy donors with and without *IgH/c-myc* translocation using CRISPR/CAS9 technology. To facilitate selection of rare *IgH/c-myc* + cells, we introduced a CMV-driven GFP tag into the reciprocal translocation breakpoint (Fig. 5a).

First, we designed and validated gRNAs capable of introducing dsDNA breakpoints into relevant *c-myc* and *IgH regions*, selecting the most efficient gRNAs using Surveyor assay (Supplementary Fig. 3). Next, we tested whether the introduction of simultaneous dsDNA breaks in *c-myc* and *IgH* regions resulted in *IgH/c-myc* translocation. We confirmed the presence of translocations by gDNA PCR (Supplementary Fig. 4). Then, we generated and validated a ssDNA insert, designed to integrate a CMV + GFP tag into the reciprocal translocation (Fig. 5a, Supplementary Fig. 5). Next, we generated and sorted *IgH/c-myc* + LCLs with GFP expressed from the insert in the reciprocal translocation breakpoint (Fig. 5b) and confirmed the presence of translocation by gDNA PCR (Fig. 5c) by sequencing (Fig. 5d), as well as by FISH (Fig. 5e, Supplementary Fig. 6).

**Fig. 6:**
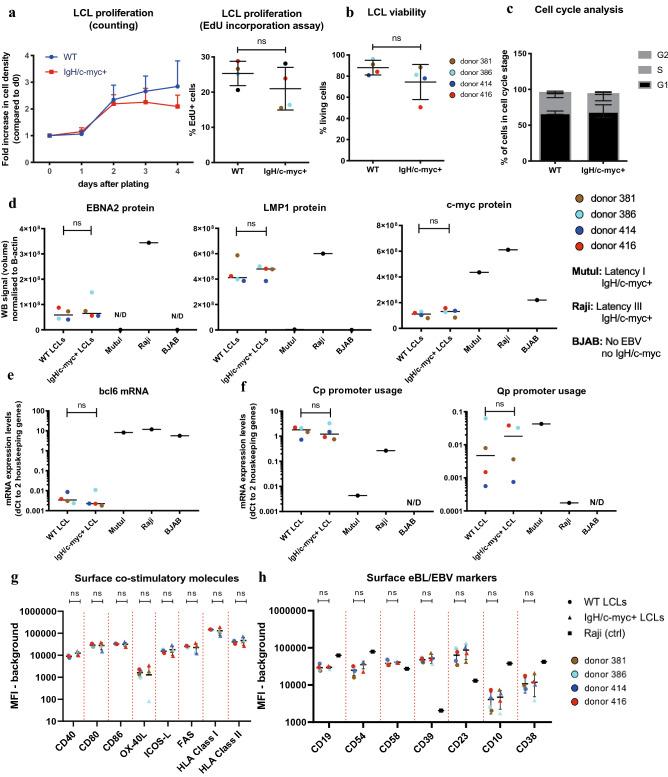
Effect of *IgH/c-myc* translocation on LCL phenotype. LCLs from four donors either with or without *IgH/c-myc* translocation were cultured at same density for 3 days and compared to assess the effect of translocation on LCL phenotype. Different symbols represent different conditions, while different colors represent different TMC donors. *p* values were calculated using paired t test. *p* > 0.05 not significant (n.s.). For flow cytometry experiments, isotype staining was used to determine positive cells. Histogram overlays for one representative donor can be found in Supplementary Fig. 8. WB images used for quantification can be found in Supplementary Fig. 6. **a** Proliferation of LCLs was assessed by counting daily with hemocytometer and by EdU incorporation assay using EdU Click-iT Flow Cytometry Kit. **b** LCL viability was assessed using Zombie viability dye. **c)** Percentage of LCLs in different cell cycle stages was measured using EdU Click-iT Flow Cytometry Kit and FxCycle dye. **d** Total protein expression was assessed using Western blotting. Western blot image was quantified and normalized to β-actin as loading control. Shown are mean ± SD of normalized volume of corresponding band. **e–f** Gene expression was determined using qRT-PCR. Shown are mean ± SD of dCt values normalized to geometric means of *TBP* and *YWHAZ.* **g**, **d** Expression of surface co-stimulatory molecules (**g**) and EBV/eBL-associated markers (**h**) was assessed using flow cytometer. Shown are mean ± SD of mean fluorescent intensity (MFI)

Finally, we tested if the presence of the translocation alone has any effect on the LCL phenotype and assessed differences in proliferation (Fig. 6a), viability (Fig. 6b) and cell cycle distribution (Fig. 6c) of *IgH/c-myc* + LCLs as compared to WT LCLs. We could not detect any significant differences between WT and *IgH/c-myc* + LCLs in RNA (Figure S8a, Fig. 6e) and protein (Fig. 6d, Supplementary Fig. 7) levels of any of the eBL/EBV growth program markers tested nor on Cp/Qp promoter usage (Fig. 6f). Furthermore, we did not observe any effect of translocation alone on expression of co-stimulatory molecules, involved in T cell/LCL interaction (Fig. 6g, Supplementary Figure S8b) or on expression of surface markers, commonly associated with EBV or eBL phenotype (Fig. 6h, Supplementary Figure S8c).

Overall, our observations are in line with the established premise that the translocation alone is not sufficient for eBL development.

### *IgH/c-myc* + pre-eBL cells do not escape viability suppression by CD4 + T cells

**Fig. 7:**
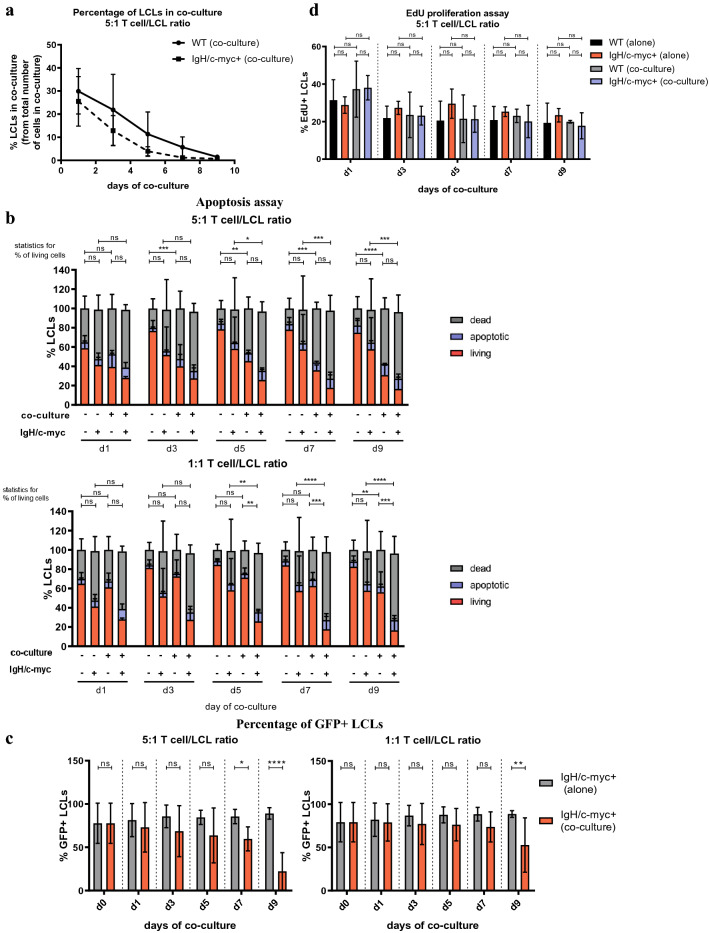
Effect of CD4 + T cells on proliferation and viability of *IgH/c-myc* + LCLs LCLs either with or without *IgH/c-myc* translocation were cultured for 9 days either alone or in co-culture with various ratios of expanded autologous CD4 + T cells activated using anti-CD3/CD28 beads. At given time points, cells were harvested, stained and analyzed using flow cytometer. LCLs were pre-gated based on CD19 expression. Shown are mean ± SD of mean percentage of positive cells from three TMC donors. *p* values were calculated using two-way ANOVA with Tukey’s test for multiple comparisons. *p* > 0.05 not significant (n.s.), *p* < 0.1*, *p* < 0.01**, *p* < 0.001***, *p* < 0.0001****. Repeats with 1:1 T cell/LCL ratios can be found in Supplementary Fig. 7. **a** Mean percentage of LCLs in co-culture was determined by CD19 and CD4 staining. **b** Mean percentage of dead, apoptotic and living LCLs was measured using Annexin V stain and Zombie Viability stain. **c** Mean percentage of GFP + LCLs. **d** Mean percentage of EdU + LCLs was measured using Click-iT Flow Cytometry Kit

**Fig. 8:**
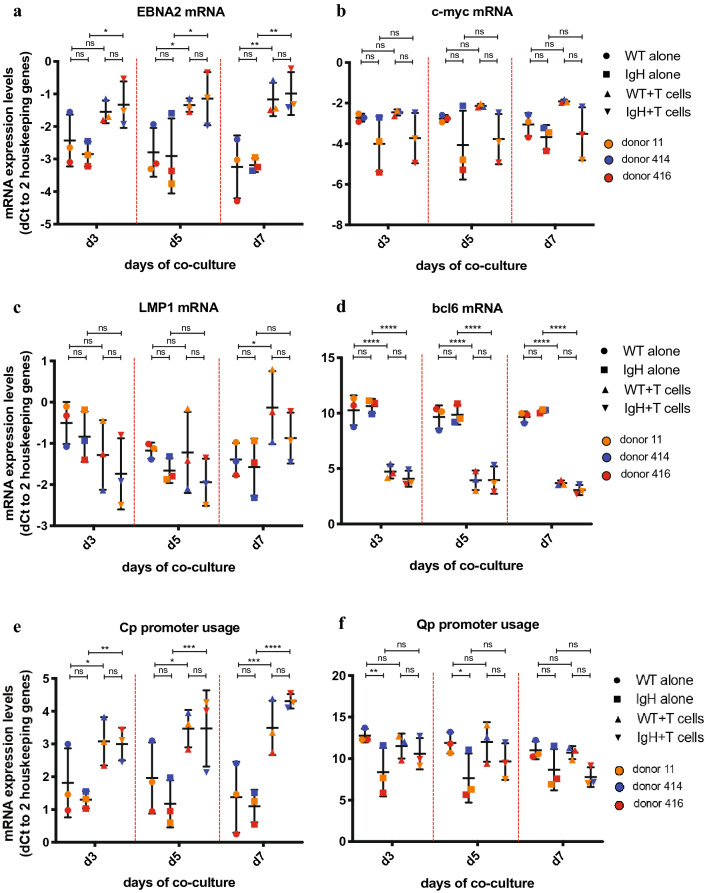
Effect of CD4 + T cells on gene expression in *IgH/c-myc* + LCLs. LCLs either with or without *IgH/c-myc* translocation were cultured for 7 days either alone or in co-culture with various ratios of expanded autologous CD4 + T cells activated using anti-CD3/CD28 beads. At given time points, cells were harvested and LCLs were isolated using CD19 + beads and autoMACS. Gene expression was determined using qRT-PCR. Shown are mean ± SD of dCt values normalized to geometric means of *TBP* and *YWHAZ*. Different symbols represent different conditions, while different colors represent different TMC donors. *p* values were calculated using two-way ANOVA with Tukey’s test for multiple comparisons. *p* > 0.05 not significant (n.s.), *p* < 0.1*, *p* < 0.01**, *p* < 0.001***, *p* < 0.0001****

Next, we used our translocation model to test whether *IgH/c-myc* + LCLs survive the interaction with CD4 + T cells better than WT LCLs. Thus, we cultured parental WT and offspring *IgH/c-myc* + LCLs in the presence of CD4 + T cells and assessed changes in proliferation, viability and mRNA/protein levels of several crucial eBL and EBV markers. While CD4 + T cells outgrew both WT and *IgH/c-myc* + LCLs, they tended to outgrow *IgH/c-myc* + LCLs faster than WT LCLs at both 5:1 (Fig. 7a) and 1:1 E/T ratio (Supplementary Fig. 8). At a 5:1 ratio, CD4 + T cells suppressed the viability of WT and *IgH/c-myc* + LCLs to a similar extent, while at a 1:1 ratio, CD4 + T cells had a stronger negative effect on viability of *IgH/c-myc* + LCLs as compared to WT LCLs (Fig. 7b). Thus, CD4 + T cells affected *IgH/c-myc* + LCLs at both a “low dose” and “high dose” E/T ratio, while the viability of WT cells was strongly reduced only at “high dose” ratio. Notably, we could also see a reduced percentage of GFP + cells among living cells in co-culture, which was more profound at 5:1 E/T ratio (Fig. 7c). There were no effects of CD4 + T cells on LCL proliferation (Fig. 7d, Supplementary Fig. 8) in co-culture or cell cycle distribution (Supplementary Fig. 8), neither on *IgH/c-myc* + LCLs nor on WT LCLs. Finally, there was no difference in CD4 + T cell effect on WT LCLs and *IgH/c-myc* + LCLs in expression of any EBV gene (EBNA2, LMP1, Cp or Qp promoter usage) or eBL markers (*c-myc* and *bcl6*) measured (Fig. 8).

Overall, our data imply that *IgH/c-myc* + LCLs are more susceptible to CD4 + T-cell-induced suppression of viability than WT LCLs.

## Discussion

Evidence accumulates that CD4 + T cells are involved in the multi-step development of eBL, but the pathogenic role of CD4 + T cells remains enigmatic. Here, we unprecedently show that CD4 + T cells are present in primary eBL tumor tissues and that CD4 + T cells on the one hand kill pre-eBL cells in vitro and on the other hand can initiate crucial EBV Latency III to Latency I switch, which supposedly takes place early in eBL development, supporting the survival of EBV-infected pre-eBL cells by lowering their immune recognition. Furthermore, while we show that the mere presence of the characteristic *IgH/c-myc* translocation does not suffice to escape CD4 + T-cell-mediated killing in vitro, the CD4 + T-cell-mediated suppression of the Latency III program in vivo may allow cells harboring the *IgH/c-myc* translocation and additional mutations, to evade immune suppression and eventually proliferate by means of deregulated *c-myc* activity, resulting in neoplasia.

A key finding of our study is the first-time demonstration of the presence of CD4 + T cells in primary eBL tissue. They may either have been present at the emergence and propagation of the eBL cells or have infiltrated the neoplastic cells after having been attracted by antigen, or both. Since CD4 + T cells, depending on their subpopulation nature, exert either helping or suppressing/cytotoxic functions, their impact on eBL cells or their precursors may be dichotomous as well.

Another key finding of our study is the demonstration that polyclonal activated CD4 + T cells suppress the viability and thereby the outgrowth of autologous pre-eBL cells. Reduced viability is further confirmed by reduction in the percentage of GFP + LCLs upon co-culture with CD4 + T cells, as GFP signal is downregulated in dying cells [39]. The exact mode of LCL cell death in our co-culture system is unclear. We did not observe changes in the relative proportion of apoptotic cells over time, suggesting that alternative cell death pathways might be involved. CD4 + T-cells-mediated loss of EBNA2 expression in LCLs offers alternative explanation, as EBNA2 confers anti-apoptotic functions and its loss was shown to reduce the viability of LCLs [40]. These data indicate that CD4 + T cells can exhibit suppressive or cytotoxic functions, in agreement with previous reports [26–29].

A third key finding in this study is our demonstration that CD4 + T cells can promote eBL emergence indirectly by inducing a switch to a more eBL-like phenotype in autologous EBV-infected pre-eBL cells resulting in immune escape. This is achieved, at least in part, through changes in expression of EBNA2, a transcription factor crucial for Latency III program, and *bcl6*, a key marker of eBL. Specifically, we show CD4 + T-cell-mediated reduction in the Cp promoter usage in LCLs and a consecutive reduction in EBNA2 expression, resulting in switching away from the dominant EBV Latency III program. As we did not see a reduction in LMP1 levels, the switch to Latency I program in our in vitro system was incomplete. This can be explained by lack of Tfh cells in our system, which can induce LMP1 suppression in LCLs via production of IL-21 [32]. Therefore, it is possible that during autologous CD4 + T cells/pre-eBL cell interaction in vivo*,* in the presence of Tfh and IL-21, full Latency III to Latency I switch does take place. Furthermore, CD4 + T cells in our co-culture are activated using polyclonal anti-CD3/CD28 beads, rendering the effect of CD4 + T cells on LCLs independent on expression of HLA Class II molecules. As EBV latency [41] and *c-myc* activity [42] are known to affect expression of HLA Class II molecules on B cells, it is possible that the in vivo effect of relatively rare EBV-specific CD4 + T cells would differ from the effect observed in our in vitro system. On the other hand, polyclonal expansion mimics the effect of non-specifically chronically stimulated CD4 + T cells, found in eBL-associated chronic malaria [37], and obviates outgrowing EBV-specific CD4 + T cells lines, which would artificially skew the phenotype and antigen specificity of CD4 + T cell pool in our in vitro system.

While CD4 + T cells suppress outgrowth of “wild-type” EBV-infected B cells, we needed to consider the possibility of rare EBV-infected B cells with *IgH/c-myc* translocation interacting with CD4 + T cells. Indeed, we hypothesized that EBV-infected B cells with *IgH/c-my*c translocation could switch to the alternative growth program and survive the interaction with CD4 + T cells better than wild-type EBV-infected B cells. Therefore, we established a novel *IgH/c-myc* translocation model. It offers a number of advantages over existing models relying on either *c-myc* overexpression or stable transfection with *c-myc/IgH*-containing plasmids [24, 25]. Firstly, we induce the translocation between endogenous *c-myc* and *IgH* genes, rather than another copy of the *c-myc* gene. Secondly, the existing epigenetic landscape is maintained. Thirdly, all relevant enhancers/regulators remain in place and at physiological distance from the *c-myc* gene. Finally, by placing the GFP tag into reciprocal translocation breakpoint, rather than into the *IgH/c-myc* translocation of interest, we avoid introducing a strong promoter into the vicinity of the *c-myc* gene. Notably, the introduction of *IgH/c-myc* translocation did not induce significant changes in LCL cell phenotype, considered as surrogate for pre-eBL. This is not surprising, as the literature suggests that EBV Latency III program is dominant over *c-myc* growth-program [24, 25].

Our final key observation is that the mere presence of *IgH/c-myc* translocation does not protect autologous EBV-infected B cells from CD4 + T-cell-mediated killing, but rather makes them more susceptible to it. This could be explained by the fact that *IgH/c-myc* + LCLs have a higher capacity for *c-myc* expression due to the presence of the translocation. In the absence of CD4 + T cells, EBNA2 would maintain a moderate level of *c-myc* expression [20, 43], suppress excessive *c-myc* expression from IgH/c-myc translocation [44] and provide some protection from *c-myc*-mediated apoptosis [40] in both WT and *IgH/c-myc* + LCLs. In the presence of CD4 + T cells, which suppress EBNA2 in LCLs, *IgH/c-myc* + LCLs become much more susceptible to apoptosis, as compared to WT LCLs, due to their capacity to express higher levels of *c-myc* from the translocated chromosome. Cells that express high levels of *c-myc* would die before the mRNA analysis, which may explain the observed lack of changes in *c-myc* levels in *IgH/c-myc* + LCLs in the presence of CD4 + T cells. Furthermore, LCLs already express high levels of *c-myc* and even small, undetectable changes in *c-myc* levels, may have strong physiological effect [45]. Fully established eBL cells, in contrast, harbor additional mutations, such as ID3 and GNA13 [5], which may protect them from *c-myc* overexpression-induced apoptosis, while allowing the benefit from *c-myc*-mediated enhancement of proliferation. Several clones of EBNA2-inducible/*c-myc*-inducible cell line were shown to survive and proliferate in a *c-myc*-dependent manner in the absence of EBNA2 after a several-week-long lag in proliferation [25], further supporting our hypothesis.

In conclusion, CD4 + T cells in eBL likely contribute to eBL pathogenesis by exhibiting a dichotomous impact through initiation of EBV Latency III to Latency I switch, thereby reducing EBV-infected B cell survival on the one hand, but increasing their survival by reducing their recognition as EBV-infected by immune cells, possibly followed by outgrowth of *IgH/c-myc*-translocated cells on the other hand. Our model of B cells harboring the *IgH/c-myc* translocation will be instrumental for further studies on eBL pathogenesis. Since the method of its generation can easily be applied to engineer other clinically relevant translocations, it may thus serve to delineate novel therapies.

## Supplementary Information

Below is the link to the electronic supplementary material.

Supplementary file1 (PDF 2324 kb)

Supplementary Fig. 9 Effect of CD4+ T cells on c-myc and bcl6 expression in LCLs LCLs were cultured for 7 days either alone or in co-culture with various ratios of expanded autologous CD4+ T cells activated using anti-CD3/CD28 beads. At given timepoints cells were harvested and LCLs were isolated using CD19+ beads and AutoMACS. Different symbols represent different conditions, while different colors represent different TMC donors. P-values were calculated using two-way Anova with Sidak’s test for multiple comparisons. p>0.05 not significant (n.s.), p<0.1*, p<0.01**, p<0.001***. WB images used for quantification can be found in Supplementary Figure 1. a) Total c-myc protein expression was assessed using Western blotting. Western blot image was quantified and normalized to -actin as loading control. Shown are mean SD of normalized volume of c-myc band. b) c-myc and bcl6 expression was determined using qRT-PCR. Shown are mean SD of dCt values normalized to geometric means of TBP and YWHAZ. (EPS 316 kb)

## Abbreviations

AID: Activation-induced cytidine deaminase
cRPM: RPMI medium
CAS9: CRISPR-associated protein 9
CMV: Cytomegalovirus
CRISPR: Clustered regularly interspaced short palindromic repeats
E/T ratio: Effector/target ratio
eBL: Endemic Burkitt lymphoma
EBNA: Epstein–Barr nuclear antigen
EBV: Epstein–Barr virus
EdU: 5-Ethynyl-2'-deoxyuridine
FISH: Fluorescence in situ hybridization
gDNA: Genomic DNA
GFP: Green fluorescent protein
IHC: Immunohistochemistry
LCL: Lymphoblastoid cell line
LMP: Latent membrane protein
LN: Lymph node
qRT-PCR: Quantitative real-time polymerase chain reaction
Tfh: Follicular T helper
TMC: Tonsillar mononuclear cell
