# Supplementary material for: CD4 + T cells are found within endemic Burkitt lymphoma and modulate Burkitt lymphoma precursor cell viability and expression of pathogenically relevant Epstein–Barr virus genes
Source: Cancer Immunol Immunother. 2021 Oct 19;71(6):1371–92. doi: 10.1007/s00262-021-03057-5 (PMC9123076; doi:10.1007/s00262-021-03057-5)
Supplement: Supplementary file 1 — Supplementary file1 (PDF 2324 kb) [file 262_2021_3057_MOESM1_ESM.pdf]

Supplementary Figure 1. Effect of CD4+ T cells on LCL EBV Latency stage and EBV gene expression. Western blot images

Western blot images for three donors used for band quantification for graphs in Figure 3 and Supplementary Figure 10

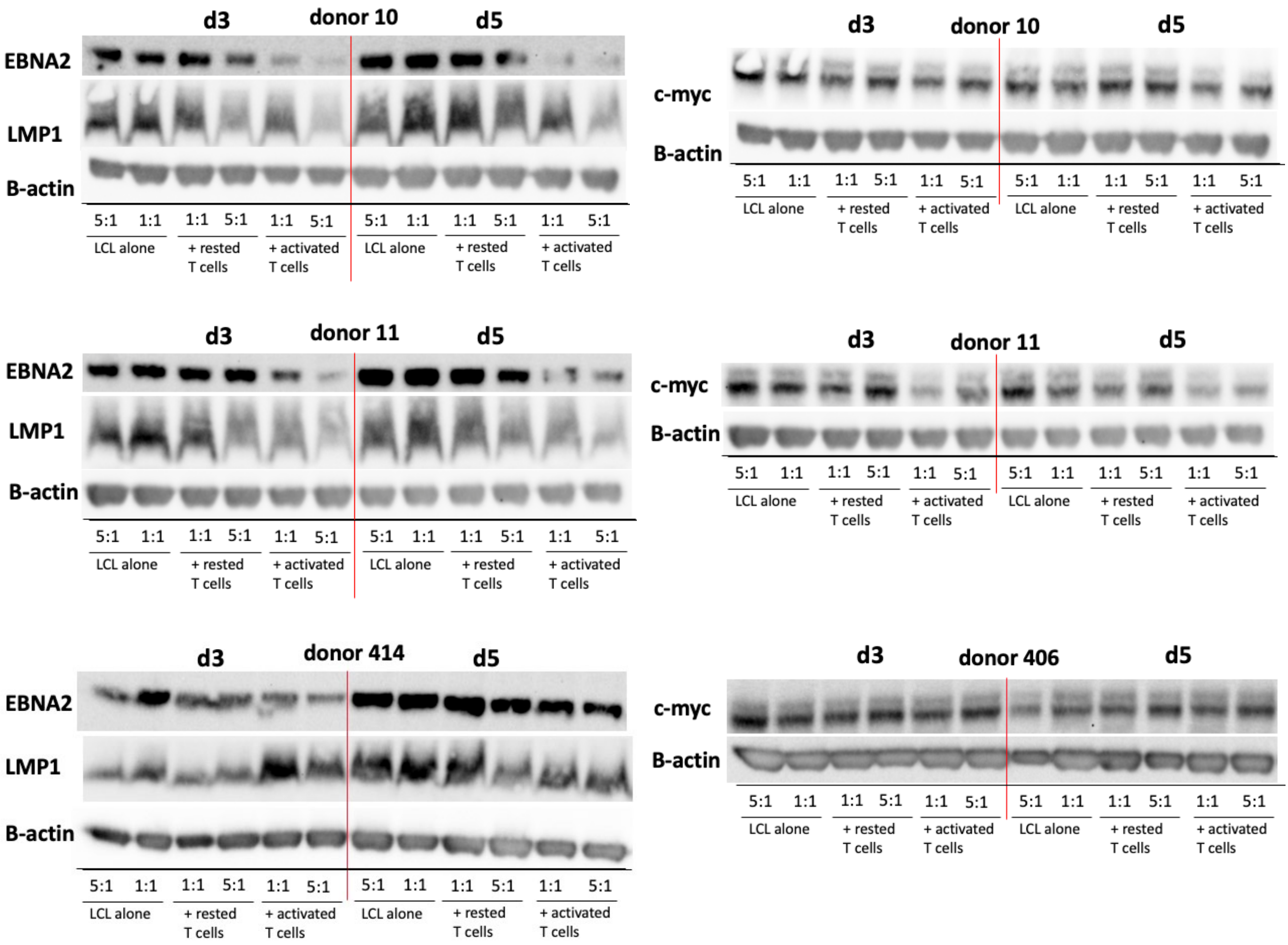

Supplementary Figure 2. Expression of co-stimulatory molecules and T-helper associated markers in in-vitro T cell-LCL co-culture system. Histogram overlays

Histogram overlays for one representative donor for Figures 4a, b (**S2a**), Figures 4c, d (**S2b**) and Figures 4e, f (**S2c**).

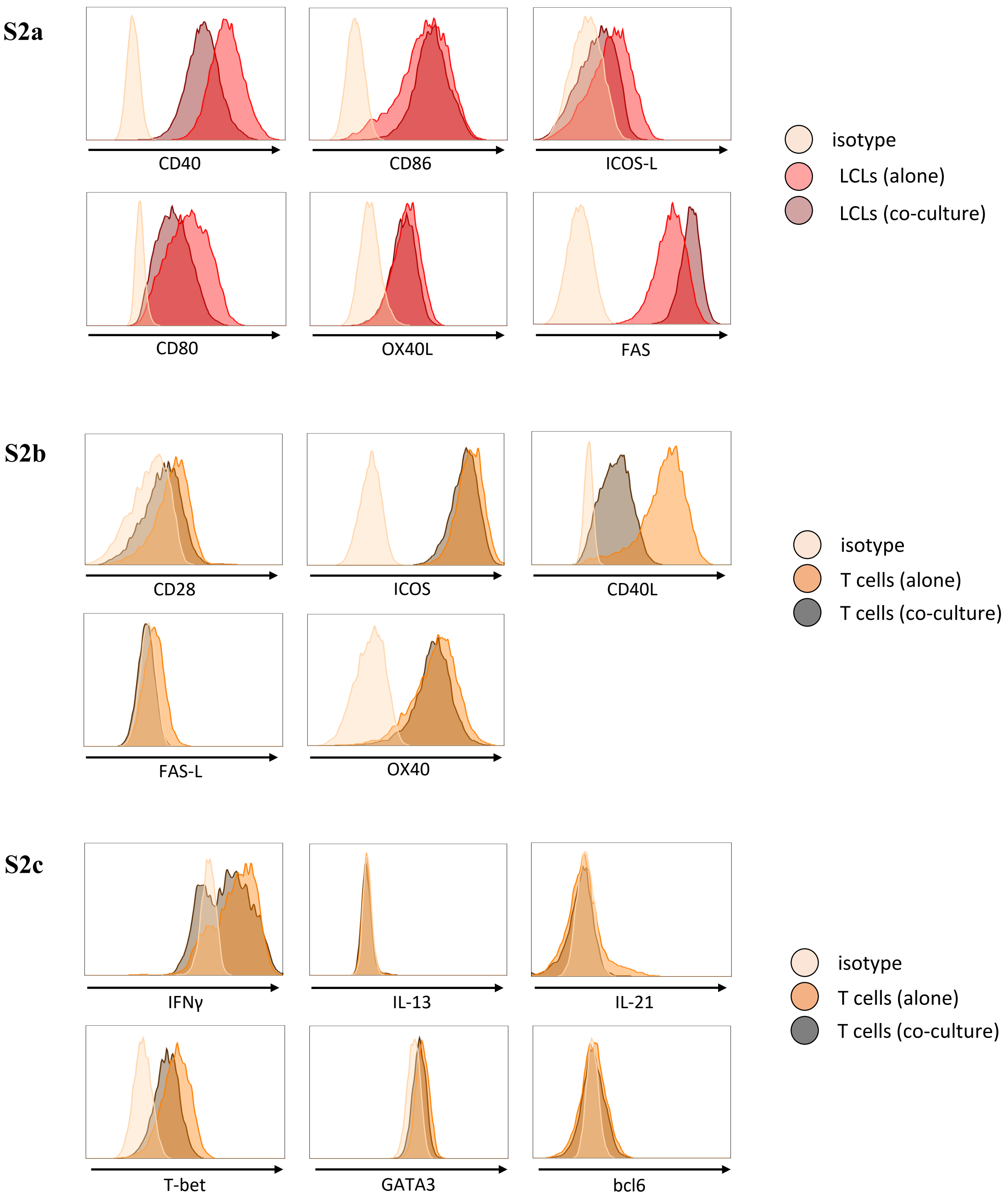

Supplementary Figure 3. Selection and validation of gRNAs for introduction of *IgH/c-myc* translocation.
a) Overview of target locations for gRNAs targeting *IgH* and *c-myc* regions. **b)** LCL line was electroporated with *IgH* *t*argeting or *c-myc* targeting RNPs. Cells were cultured for 3 days post electroporation, then gDNA was isolated and assessed for presence of mutations using Surveyor Assay kit.

S3a

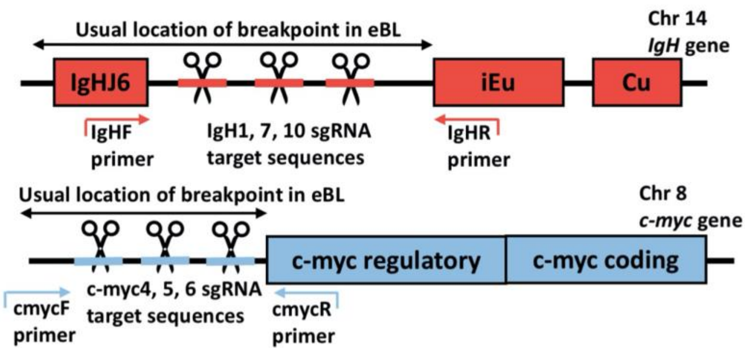

S3b

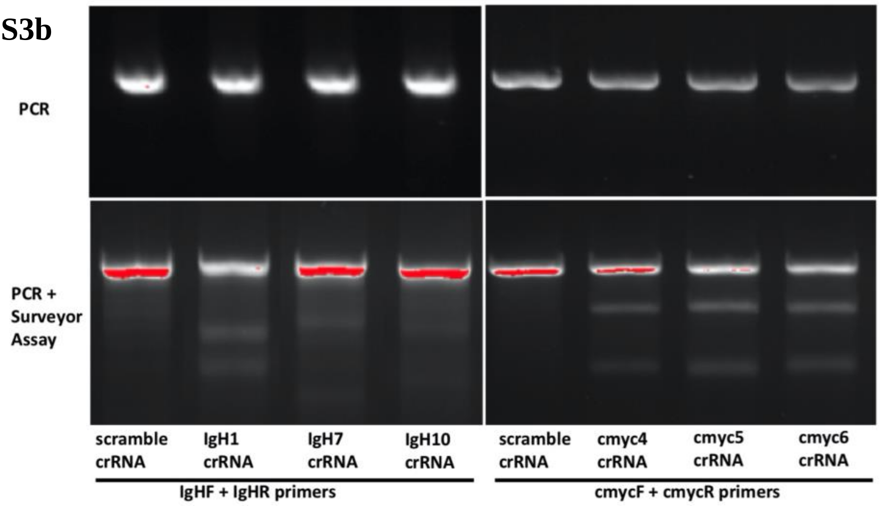

Supplementary Figure 4. Introduction of *IgH/c-myc* translocation using *IgH* and *c-myc* targeting CAS9 RNPs. **a)** Overview of gDNA PCR design used to detect *IgH/c-myc* translocations. **b)** LCL line was electroporated with RNPs targeting both *IgH* and *c-myc* regions. Cells were cultured for 3 days post electroporation, then gDNA was isolated and assessed for presence of translocation using gDNA PCR. **c)** Individual bands were cut out from the agarose gel, cloned into vector and sequenced.

S4a

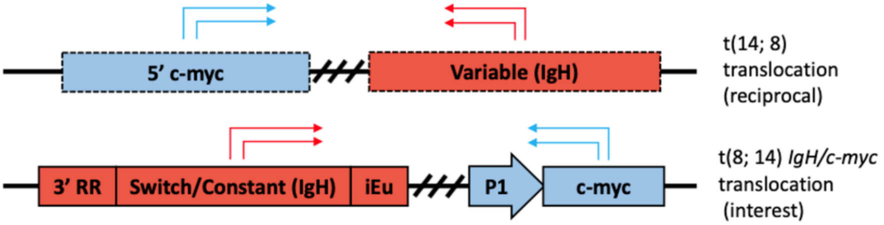

S4b

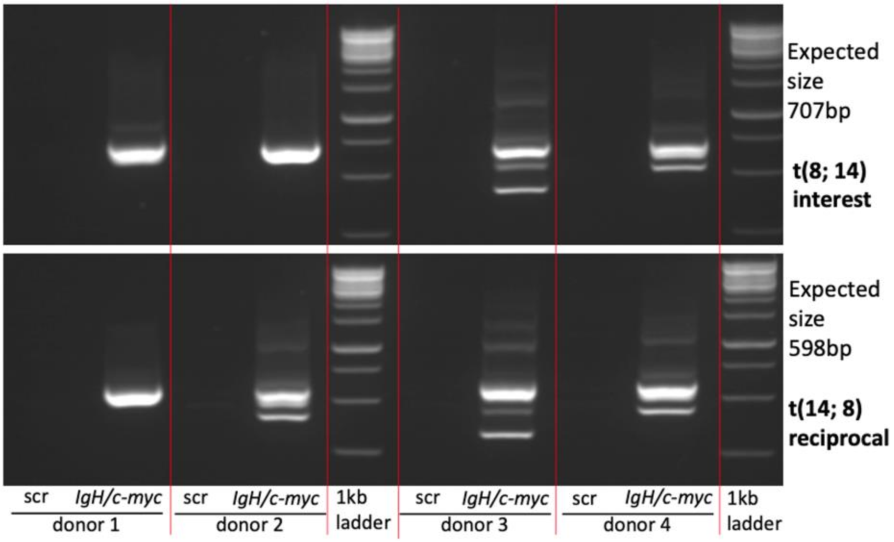

S4c

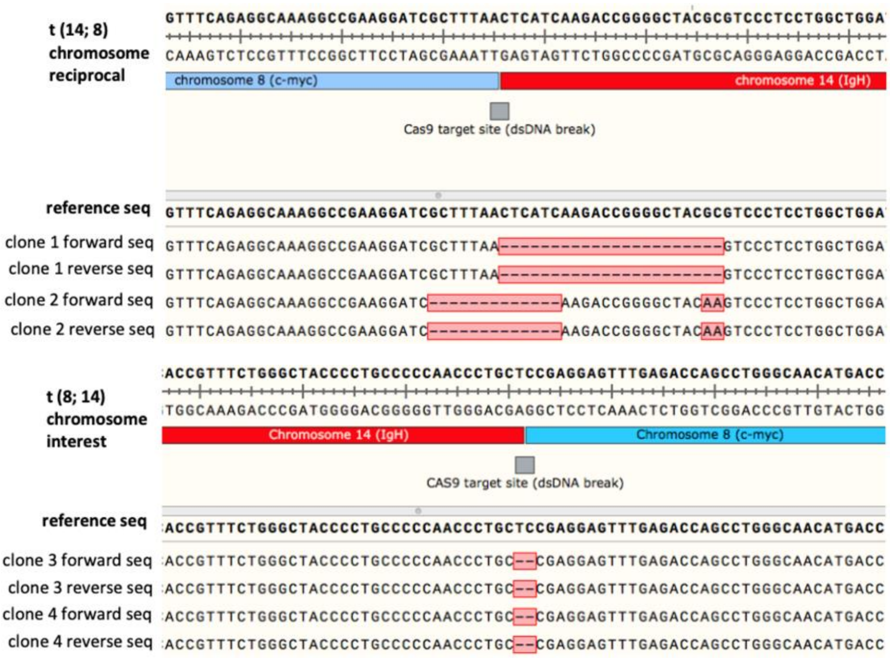

Supplementary Figure 5. Generation of ssDNA insert for introduction of GFP tag into *IgH/c-myc* translocation.
a) Overview of plasmid used for generation of ssDNA insert. ssDNA was generated from PCR product, size and sequence were confirmed by gel electrophoresis **(b)** and Sanger sequencing **(c).**

S5a

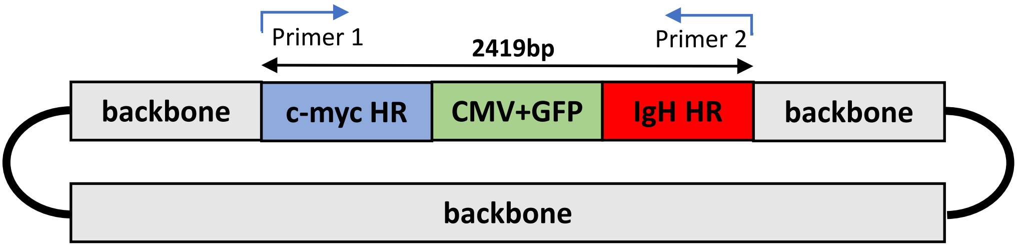

S5b

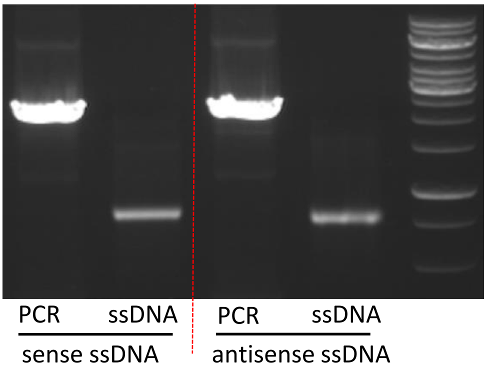

S5c

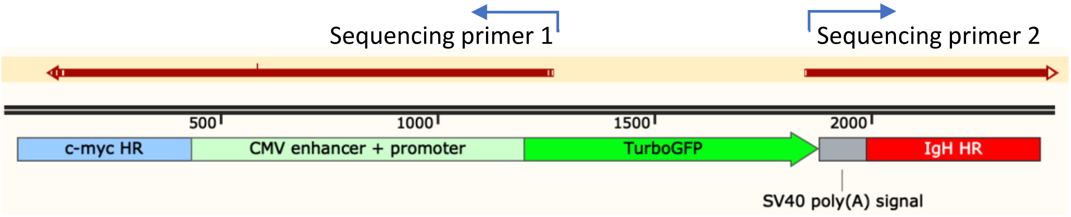

Supplementary Figure 6. Confirmation of presence of *IgH/c-myc* translocation in CRISPR/CAS9 edited LCL lines

a) Overview of gDNA PCR design used to detect *IgH/c-myc* translocations. **b-d)** LCLs from 4 donors either with or without *IgH/c-myc* translocation were cultured for 3 days. **b)** Percentage of GFP+ cells was determined by flow cytometry prior to harvesting. **c)** gDNA was isolated and the presence of the translocation was assessed by gDNA PCR. **d)** Dual probe FISH for *IgH/c-myc* translocation was performed. Representative images for each donor for both "wild type" and *IgH/c-myc*+ LCLs are shown.

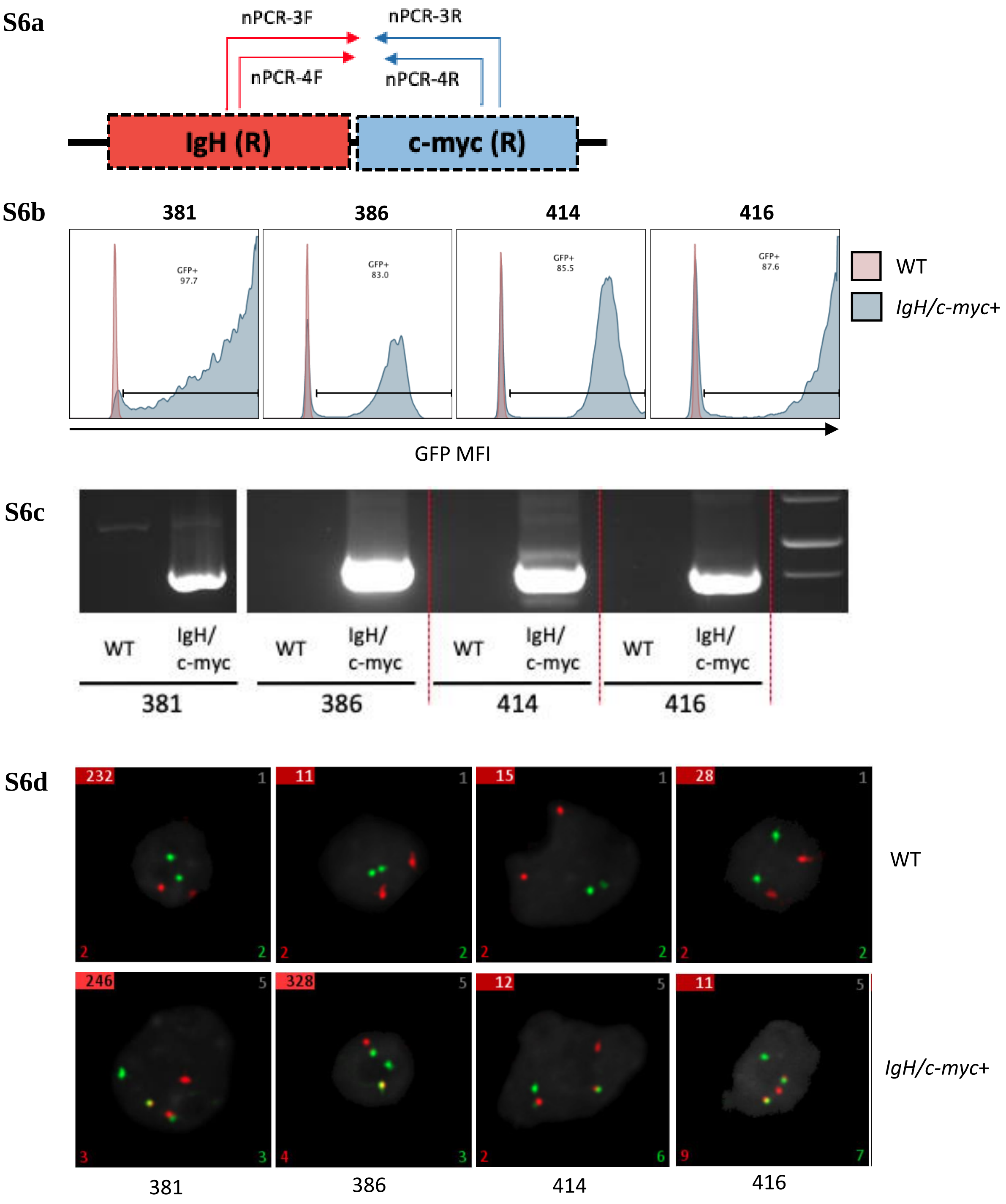

Supplementary Figure 7. Effect of *IgH/c-myc* translocation on LCL gene expression. Western blot images
Western blot images for four donors used for band quantification used for graphs in Figure 6.

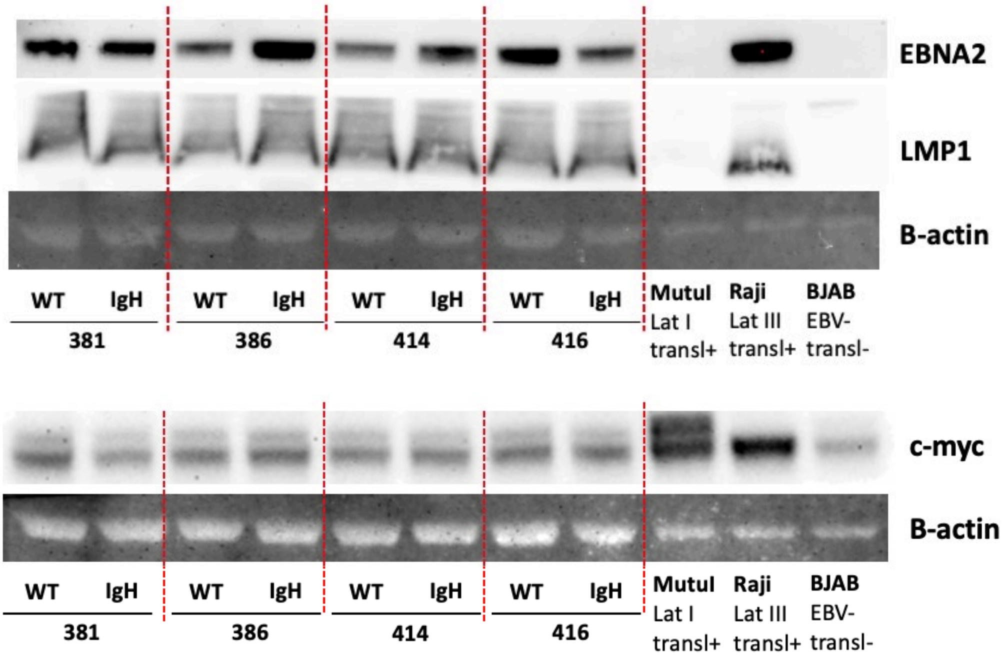

Supplementary Figure 8. Effect of *IgH/c-myc* translocation on LCL phenotype

LCLs from 4 donors either with or without *IgH/c-myc* translocation were cultured at same density for 3 days and compared to assess the effect of translocation on LCL phenotype. Different symbols represent different conditions, while different colors represent different TMC donors. P-values were calculated using paired t-test. p>0.05 not significant (n.s.). For flow cytometry experiments, isotype staining was used to determine positive cells.

a) Gene expression was determined using qRT-PCR. Shown are mean ± SD of dCt values normalized to geometric means of *TBP* and *YWHAZ*. **b, c)** Histogram overlays for one representative donor for Figure 6g (**S2b**) and Figure 6h (**S2c**)

S8a

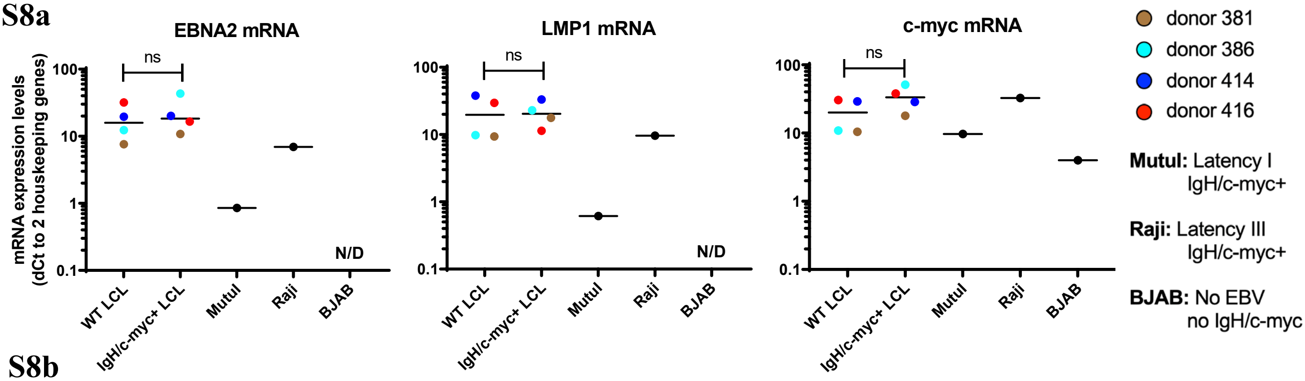

S8b

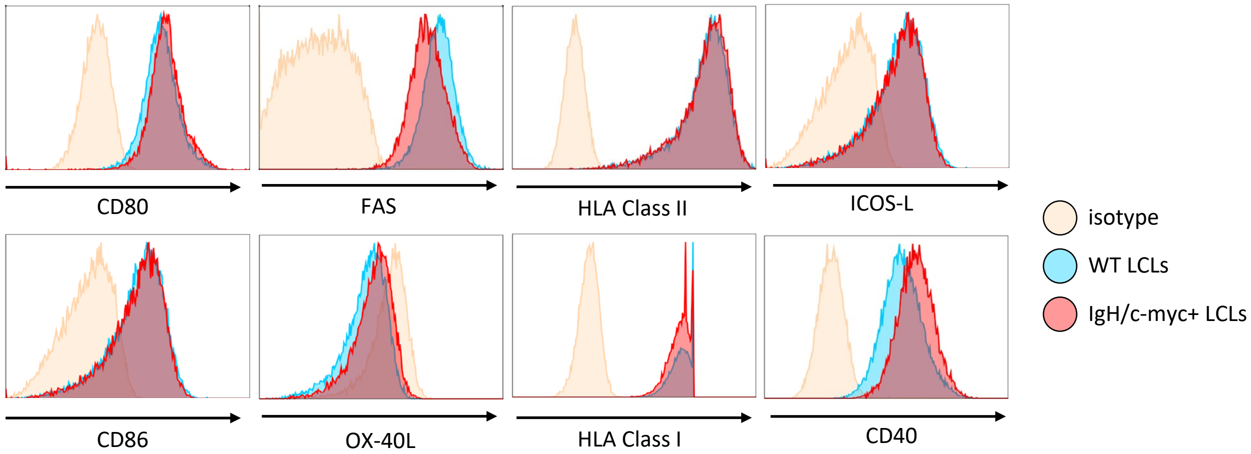

S8c

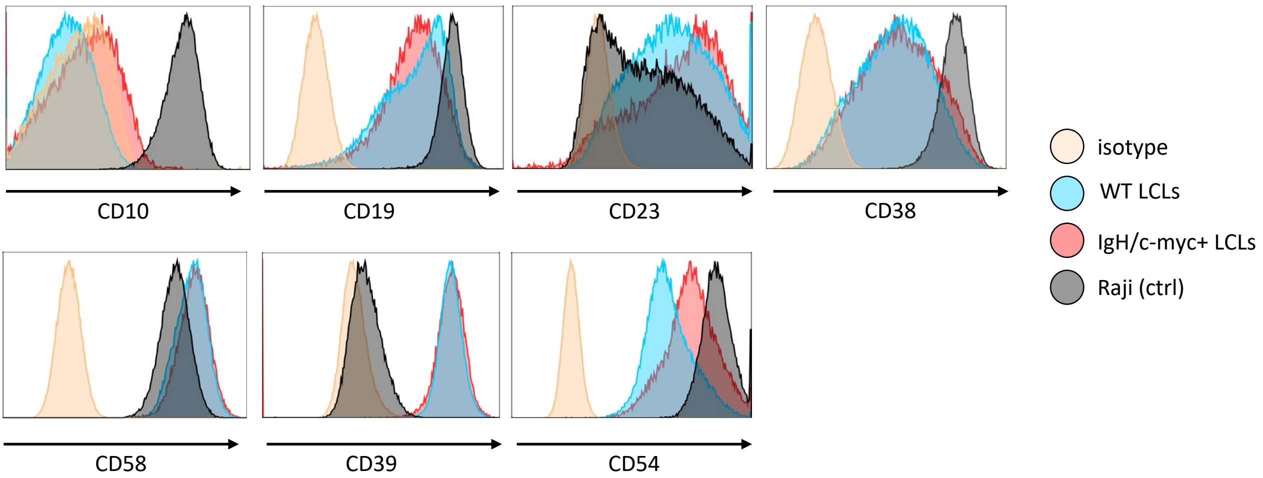

Supplementary Figure 9. Effect of CD4+ T cells on proliferation and viability of *IgH/c-myc*+ LCLs

LCLs either with or without *IgH/c-myc* translocation were cultured for 9 days either alone or in co-culture with various ratios of expanded autologous CD4+ T cells activated using anti-CD3/CD28 beads. At given timepoints cells were harvested, stained and analysed using flow cytometer. LCLs were pre-gated based on CD19 expression. Shown are mean ± SD of mean percentage of positive cells from 3 TMC donors. P-values were calculated using two-way Anova with Tukey's test for multiple comparisons. $p>0.05$ not significant (n.s.), $p<0.1$*, $p<0.01$**, $p<0.001$***, $p<0.0001$****.

a) Mean percentage of LCLs in co-culture was determined by CD19 and CD4 staining. **b)** Mean percentage of EdU+ LCLs was measured using Click-iT Flow Cytometry kit. **c)** Mean percentage of LCLs in different cell cycle stages was measured using EdU Click-iT Flow cytometry kit and FxCycle dye.

S9a

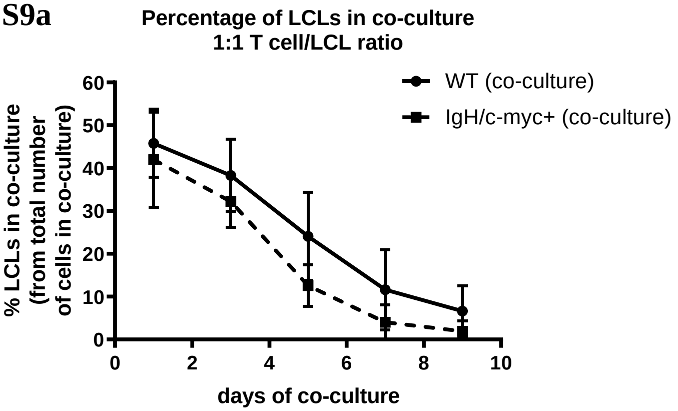

S9b

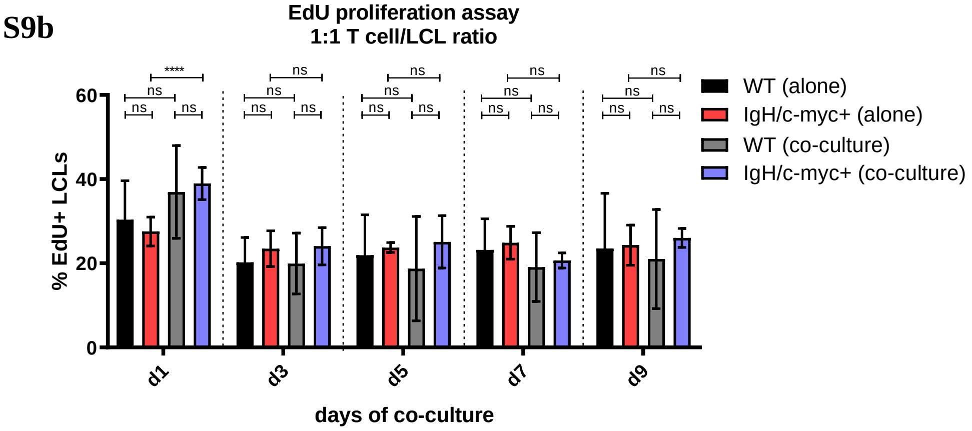

S9c

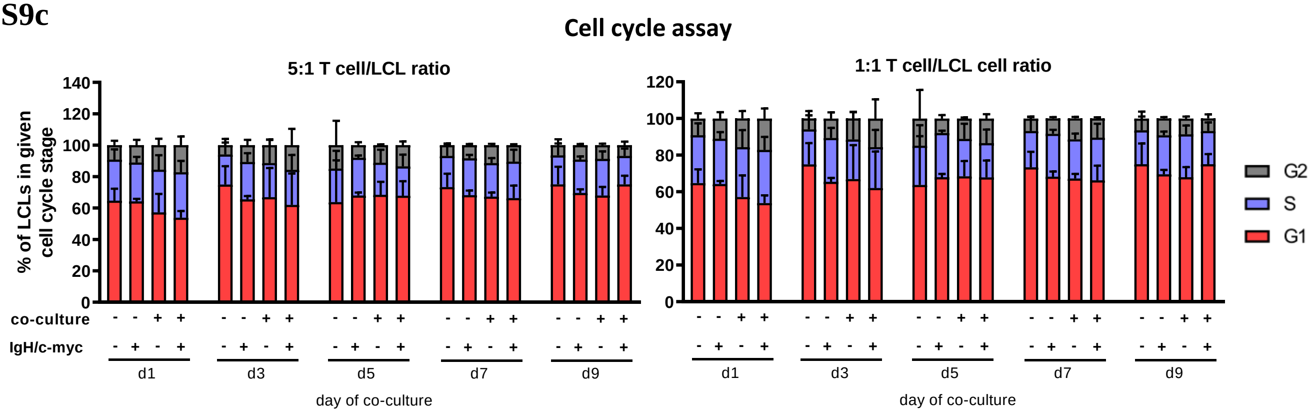

Supplementary Figure 10. Effect of CD4+ T cells on *c-myc* and *bcl6* expression in LCLs
LCLs were cultured for 7 days either alone or in co-culture with various ratios of expanded autologous CD4+ T cells activated using anti-CD3/CD28 beads. At given timepoints cells were harvested and LCLs were isolated using CD19+ beads and AutoMACS. Different symbols represent different conditions, while different colors represent different TMC donors. P-values were calculated using two-way Anova with Sidak's test for multiple comparisons. $p>0.05$ not significant (n.s.), $p<0.1$*, $p<0.01$**, $p<0.001$***. WB images used for quantification can be found in Supplementary Figure 1. **a)** Total *c-myc* protein expression was assessed using Western blotting. Western blot image was quantified and normalized to β-actin as loading control. Shown are mean ± SD of normalized volume of *c-myc* band. **b)** *c-myc* and *bcl6* expression was determined using qRT-PCR. Shown are mean ± SD of dCt values normalized to geometric means of *TBP* and *YWHAZ.*

S10a

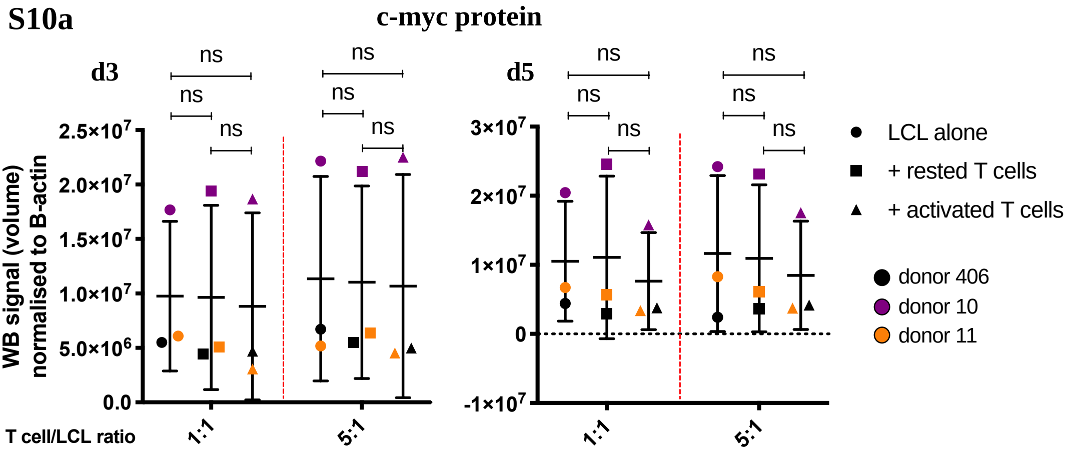

S10b

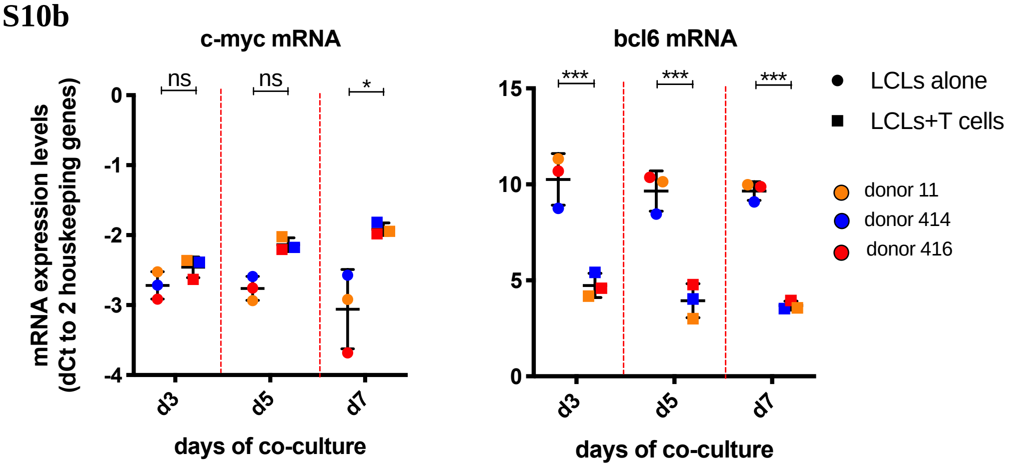

Supplementary Table 1. Part 1. Details eBL patients used for IHC stainings

**Case**	**Gender**	**Age (YRS) at diagnosis**	**Topography**	**Morphology**		FISH
				**Cell size**	**Starry-sky (NP) Not Present (P) Present**	**t8; 14 (c-myc translocation)**
**BL 1**	M	45	DUODENAL LESION	MEDIUM	NP	POSITIVE t(8;14)
**BL 2**	F	32	LYMPH NODE	MEDIUM	P	NEGATIVE (8;14); NEGATIVE c-MYC translocation
**BL 3**	M	70	PAROTID GLAND	MEDIUM	P	POSITIVE t(8;14)
**BL 4**	F	UNSPECIFIED	LEFT AXILLARY NODE	MEDIUM	P	POSITIVE t(8;14)
**BL 5**	M	39	R AXILLARY NODE	MEDIUM	P	POSITIVE t(8;14)
**BL 6**	M	26	LYMPH NODE	MEDIUM	P	POSITIVE c-MYC rearrangement, NEGATIVE BCL2 & BCL6 rearrangement
**BL 7**	F	30	FALLOPIAN TUBE	MEDIUM	P	POSITIVE t(8;14)
**BL 8**	F	29	L AXILLARY NODE	MEDIUM	P	POSITIVE t(8;14)
**BL 9**	M	29	AXILLARY NODE	MEDIUM	P	NEGATIVE t(8;14) NEGATIVE c-MYC rearrangement
**BL10**	F	32	PAROTID GLAND	MEDIUM	P	UNSUCCESSFUL FISH
**BL 11**	M	42	LEFT CERVICAL NODE	MEDIUM	P	UNSUCCESSFUL
**BL 12**	M	32	INGUINAL NODE	MEDIUM	P	POSITIVE C-MYC REARRANGEMENT
**BL 13**	M	40	R AXILLARY LYMPH NODE	MEDIUM	P	POSITIVE t(8;14)

Supplementary Table 1. Part 2. Details eBL patients used for IHC stainings

Case	Immunochemistry (P) - positive (>30% staining)							
	**CD20**	**CD10**	**Bcl6**	**bcl2**	**Ki67**	**TdT**	**MuM1**	**Cyclin D1**
**BL 1**	P	P	P	N	100%	NP	N	
**BL 2**	P	P	P	N	100%	NP	N	
**BL 3**	P	P	P	N	100%		N	
**BL 4**	P	P	P	N	100%	N	N	
**BL 5**	P	P	P	N	100%	NP	N	
**BL 6**	P	P	P	N	100%	NP	N	
**BL 7**	FOCAL P	P	P	N	100%	**N**	P	CD138 NEG
**BL 8**	P	P	P	N	100%	N	NP	
**BL 9**	P	P	P	N	100%	N	NP	
**BL10**	P	P	P	N	100%	N	N	
**BL 11**	P	P	P	N	100%	N	N	N
**BL 12**	P	P	P	N	100%	N	N	N
**BL 13**	P	P	P	N	100%	N	N	NP

Supplementary Table 2. eBL tumour IHC data

Case	CD3	CD4	CD8
**BL 1**	2	1	2
**BL 2**	2	2	2
**BL 3**	3	1	3
**BL 4**	2	2	2
**BL 5**	2	1	2
**BL 6**	5	3	4
**BL 7**	2	2	2
**BL 8**	5	4	4
**BL 9**	3	2	3
**BL 10**	2	2	2
**BL 11**	2	2	2
**BL 12**	2	2	2
**BL 13**	2	1	3

`

**numbers in % of total infiltrate**
n/a: not available
f/n: false negative
0: negative
1: < 1%
2: 1-5%
3: 10%
4: 10-20%
5: 21-30%
